# Structure and Phylogeny of the Curly Birch Chloroplast Genome

**DOI:** 10.3389/fgene.2021.625764

**Published:** 2021-10-04

**Authors:** Konstantin A. Shestibratov, Oleg Yu. Baranov, Eugenia N. Mescherova, Pavel S. Kiryanov, Stanislav V. Panteleev, Ludmila V. Mozharovskaya, Konstantin V. Krutovsky, Vladimir E. Padutov

**Affiliations:** ^1^Forest Biotechnology Group, Branch of Shemyakin and Ovchinnikov Institute of Bioorganic Chemistry, Russian Academy of Sciences, Pushchino, Russia; ^2^Forestry Faculty, G. F. Morozov Voronezh State University of Forestry and Technologies, Voronezh, Russia; ^3^Laboratory of Genomics and Bioinformatics, Forest Research Institute, National Academy of Sciences of Belarus, Gomel, Belarus; ^4^Department of Forest Genetics and Forest Tree Breeding, George-August University of Göttingen, Göttingen, Germany; ^5^Laboratory of Population Genetics, N. I. Vavilov Institute of General Genetics, Russian Academy of Sciences, Moscow, Russia; ^6^Laboratory of Forest Genomics, Genome Research and Education Center, Institute of Fundamental Biology and Biotechnology, Siberian Federal University, Krasnoyarsk, Russia; ^7^Department of Genetics, Tree Breeding and Biotechnology, Forest Research Institute, National Academy of Sciences of Belarus, Gomel, Belarus

**Keywords:** *Betula pendula* var. *carelica*, chloroplast, curly birch, genome, microsatellites, Molecular markers, phylogeny, plastome

## Abstract

Curly birch [*Betula pendula* var. *carelica* (Merckl.) Hämet-Ahti] is a relatively rare variety of silver birch (*B. pendula* Roth) that occurs mainly in Northern Europe and northwest part of Russia (Karelia). It is famous for the beautiful decorative texture of wood. Abnormal xylogenesis underlying this trait is heritable, but its genetic mechanism has not yet been fully understood. The high number of potentially informative genetic markers can be identified through sequencing nuclear and organelle genomes. Here, the *de novo* assembly, complete nucleotide sequence, and annotation of the chloroplast genome (plastome) of curly birch are presented for the first time. The complete plastome length is 160,523 bp. It contains 82 genes encoding structural and enzymatic proteins, 37 transfer RNAs (tRNAs), and eight ribosomal RNAs (rRNAs). The chloroplast DNA (cpDNA) is AT-rich containing 31.5% of A and 32.5% of T nucleotides. The GC-rich regions represent inverted repeats IR1 and IR2 containing genes of rRNAs (*5S*, *4.5S*, *23S*, and *16S*) and tRNAs (*trn*V, *trn*I, and *trn*A). A high content of GC was found in rRNA (55.2%) and tRNA (53.2%) genes, but only 37.0% in protein-coding genes. In total, 384 microsatellite or simple sequence repeat (SSR) loci were found, mostly with mononucleotide motifs (92% of all loci) and predominantly A or T motifs (94% of all mononucleotide motifs). Comparative analysis of cpDNA in different plant species revealed high structural and functional conservatism in organization of the angiosperm plastomes, while the level of differences depends on the phylogenetic relationship. The structural and functional organization of plastome in curly birch was similar to cpDNA in other species of woody plants. Finally, the identified cpDNA sequence variation will allow to develop useful genetic markers.

## Introduction

Curly birch [*Betula pendula* var. *carelica* (Merckl.) Hämet-Ahti] is a relatively rare variety of silver birch (*B. pendula* Roth) in the *Betula* tree genus. It is famous for a unique decorative texture trait observed in its wood, which determines its economic importance as a source of valuable raw material for the craft and furniture industry (Novitskaya, 2008).

The market value of patterned wood of Karelian curly birch is much higher than that of regular wood of silver birch or downy birch (Khakimova, 2016). At the same time, the biological resources of curly birch are limited, which is associated with both its low productivity and relatively rare and scattered occurrence. Curly birch does not form pure stands and grows as single trees or groups in the second or third tier of forest stands (Vetchinnikova et al., 2013). Therefore, the conservation, sustainable exploitation, and reproduction of biological resources of the curly birch are crucial tasks that require the simultaneous use of various approaches for achieving them.

The unique decorative texture of curly birch wood is a result of abnormal xylogenesis and heritable, but its genetic mechanism has not yet been fully understood. According to its taxonomic status, curly birch is classified as a variety of silver birch, although trees with a similar wood structure were also found among downy birches (*B. pubescens* Ehrh.), as well as interspecific hybrids *B. pendula* × *pubescens*. Curly birch has high phenotypic diversity with different habitus ranging from 25-m-tall trees to small branching shrubs with short rising trunks and different levels and patterns of the decorative texture trait expression in its wood (Vetchinnikova et al., 2013). Although heritable, the expression of this trait largely depends on many external factors, including also light mode. For example, in several studies, formation of a patterned texture occurred only in conditions of intense illumination and *vice versa*—a decrease in the expression of this trait was observed because of lack of or insufficient sunlight (Vetchinnikova et al., 2013).

Curly birch can be reproduced by both seeds and vegetative cloning. However, the efficiency of seed propagation, even with controlled pollination, is low. Vegetative *in vitro* propagation allowed us to obtain planting material with reproducible morphological and anatomical parameters of wood. However, this method limits the potential variety of texture options and source material for breeding (Butorina, 1993; Vetchinnikova, 2005; Vetchinnikova et al., 2013).

It is well-known that open exposure to bright light has a significant effect on the development of abnormal growth of annual rings. It has been shown that the formation of patterned phenotypes in cloned or seed planting material of curly birch is possible only when trees are grown in open areas. Often after severe harvesting in birch forests, some of the remaining trees that apparently have a “patterned” promoting genotype begin to form abnormal wood. It was observed that a “patterned” wood can develop only at the well-lit side and disappear in the absence of the required amount of light. The photophily of curly birch was also confirmed by experimental data: plants with signs of abnormal xylogenesis die when the crowns of regular birch trees form canopy closure in the seed plantations established for growing curly birch (Vetchinnikova, 2005; Vetchinnikova et al., 2013).

Complete description of the molecular mechanisms underlying abnormal xylogenesis has not yet been reached (Moshchenskaya et al., 2017). Among factors determining the formation of patterned wood in curly birch are the lighting conditions that apparently affect the functioning of the photosynthetic apparatus localized in the chloroplasts and controlled by both chloroplast and nuclear genes. Expression of chloroplast genes can be regulated by transcription (Kusnetsov, 2018), epigenetic modifications (Choubey and Rajam, 2015), RNA editing, local structural change, and the arrangement of genes in chloroplast DNA (cpDNA). Although the structural and functional organization of the chloroplast genome (plastome) in plant organisms is highly conserved (Olmstead and Palmer, 1994), the plastome sequence is needed to better understand these genetic mechanisms and how the chloroplast genes in curly birch are associated with abnormal xylogenesis. Both nuclear and plastome sequence data are needed to associate the curly birch wood traits and other important traits with particular genes. The cpDNA markers will be also very useful for phylogenetic studies of birch species.

The main objectives of this study were to (1) sequence cpDNA and *de novo* assemble and annotate plastome of curly birch, (2) compare its structural and functional organization with other woody species, and (3) identify cpDNA polymorphisms that can be used as molecular genetic markers in further studies.

## Materials and Methods

### Plant Material

Fragments of the plant tissue culture (leaves, stems) of the curly birch clone KC06 from the *in vitro* culture collection of the Forest Research Institute of the National Academy of Sciences of Belarus were used in the study. They were grown on antibiotic-free Woody Plant Medium (WPM) (McCown and Lloyd, 1981). No bacterial or fungal contaminations were found on plant explants tested using diagnostic universal PCR primers for fungal ITS2 (White et al., 1990) and bacterial 16S genes (Baker et al., 2003).

### Chloroplast Isolation

Chloroplasts were isolated from *in vitro* plant tissue culture using differential centrifugation technique to enrich DNA samples by cpDNA (Shi et al., 2012; Vieira et al., 2014). Plant materials (15–20 mg) were homogenized in 20 ml of isolation buffer at 4°C containing 60 mM Tris, 0.4 mÌ sorbitol, 7.2 mM EDTA-Na_2_, and 0.15% 2-mercaptoethanol. The final pH of the solution was 8.1. The liquid phase (1.5 ml) of homogenate was transferred to microcentrifuge tubes (1.75 ml) and centrifuged at 200 × *g* at 4°C for 15 min. The supernatant (1 ml) was transferred to another microcentrifuge tube (1.75 ml) and centrifuged at 200 × *g* at 4°C for 20 min. The final supernatant was discarded, and the pellet with chloroplasts was resuspended in 1.5 ml of washing buffer containing 60 mM Tris, 0.4 mM sorbitol, and 5 mM EDTA-Na_2_. The resuspended pellet containing chloroplasts was centrifuged at 2,000 × *g* at 4°C for 10 min. The final pH of the solution was 8.1. The purification of chloroplasts was repeated five times.

### Isolation of Chloroplast DNA

Chloroplast DNA isolation was based on the ethanol precipitation technique. The chloroplast pellets were resuspended in 100 μl of DNA isolation buffer (*T* = 25°C) containing 2% CTAB, 0.1 M Tris, 1.4 M NaCl, and 20 mM EDTA-Na_2_. The final pH of the solution was 8.0. All samples were transferred in one microcentrifuge tube (1.75 ml), then mixed on vortex mixer (2,000 min^–1^) for 1 min and incubated in a thermostat at 65°C for 20 min. An equal volume of chloroform was added to the tube and mixed on a vortex mixer (400 min^–1^) for 10 min. Then, centrifugation was performed at 15,000 × *g* at 4°C for 20 min. The maximum possible amount of supernatant was pipetted and transferred to another 1.75-ml microcentrifuge tube, then 0.7 volume of isopropanol was added. Next, samples were mixed in a vortex mixer (400 min^–1^) for 1 min, then centrifuged at 15,000 × *g* at 4°C for 10 min. The supernatant was discarded. The DNA pellet was washed with 900 μl of 65% ethanol before being cooled to −18°C and then centrifuged at 15,000 × *g* at 4°C for 10 min. All samples were washed 2–3 times. After DNA purification, the tubes with open lids were placed in a rack to let the DNA pellet air-dry for 30–40 min at 45°C until the ethanol is evaporated. The dried precipitate was dissolved in 100 μl of deionized water in a thermostat at 45°C for 15 min. Dissolved DNA was stored at 4°C.

### Chloroplast DNA Library Preparation and Sequencing

Chloroplast DNA sequencing was performed using the Ion Torrent^TM^ PGM System and sequencing reagents (Thermo Fisher Scientific Inc., Waltham, MA, United States) according to the manufacturer’s recommended protocol for DNA libraries of 200 bp.

First, fragment DNA libraries were created (read size ≈200 bp) using the Ion Plus Fragment Library Kit (Thermo Fisher Scientific Inc., Waltham, MA, United States). Standardization of library concentrations (up to 100 pM) was achieved using the Ion Library Equalizer Kit (Thermo Fisher Scientific Inc., Waltham, MA, United States). The created libraries were used for emulsion PCR (ePCR). Master mix for ePCR was prepared using the Ion PGM Template OT2 200 Kit (Thermo Fisher Scientific Inc., Waltham, MA, United States) according to the manufacturer’s recommended protocol. The ePCR was performed using the Ion OneTouch 2 System plate thermocycler (Thermo Fisher Scientific Inc., Waltham, MA, United States). The microsphere enrichment stage was carried out using the Ion OneTouch ES automatic sample preparation system with the Ion PGM Template OT2 Solutions 200 Kit and Ion PGM Enrichment Beads (Thermo Fisher Scientific Inc., Waltham, MA, United States). The sequencing reaction was carried out using the Ion PGM System with the Ion 314 Chip v2 and Ion PGM Sequencing 200 Kit v2 kits (Thermo Fisher Scientific Inc., Waltham, MA, United States). Final sample preparation and chip loading were carried out according to the manufacturer’s recommended protocol.

### Assembly and Annotation of the Plastome

Sequencing reads were initially processed automatically using the Ion Torrent Suite Software v. 5.8 (Thermo Fisher Scientific Inc., Waltham, MA, United States). Further sequence processing and assembly were done using SPAdes v. 3.13 assembler (Bankevich et al., 2012). Plastome annotation and analysis were accomplished using online services and software packages that can be found in the public domain: CpGAVAS (Liu et al., 2012), AGORA (Jung et al., 2018), GC Content Calculator^1^, OGDraw (Lohse et al., 2007), REGRNA v.2.0 (Chang et al., 2013), MEGA v.7.0 (Kumar et al., 2016), and mVISTA (Brudno et al., 2007). The verification of the assembly was done by plotting the *B. pendula* var. *carelica* (MG966529.2) complete plastome sequence vs. plastomes of eight birch species using D-GENIES online tool (Cabanettes and Klopp, 2018).

### Search for Microsatellite Loci

Genome-wide Microsatellite Analyzing Tool (GMATo) was used to search for the SSR loci in the plastome with a threshold of a minimum of seven repeats for mononucleotide, six for dinucleotide, and five for tri-, tetra-, and more nucleotide motifs (Wang et al., 2013).

### Phylogenetic Analysis

The nucleotide sequences of the plastome tRNA genes and the whole plastomes were aligned using MAFFT v.7 software (Katoh et al., 2002). The phylogenetic analysis of the tRNA genes was conducted in MEGA7 (Kumar et al., 2016) using the maximum likelihood (ML) method based on the Tamura-Nei model. Initial trees for the heuristic search were obtained automatically by applying neighbor-joining (NJ) and BioNJ algorithms to a matrix of pairwise distances estimated using the maximum composite likelihood (MCL) approach and then selecting the topology with superior log likelihood value.

The phylogenetic analysis was done also using whole plastome sequences of eight different birch species and *Alnus glutinosa* as an outgroup species. The dendrogram was constructed by applying NJ algorithm (Studier and Keppler, 1988) and Jukes–Cantor correction method (Jukes and Cantor, 1969). The consensus tree was based on 1,000 bootstrap sets.

## Results and Discussion

### Plastome Assembly and Annotation

**FIGURE 1:**
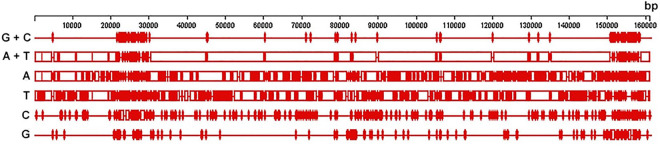
Distribution of nucleotide (A, T, C, and G), GC- and AT-rich content in the plastome of curly birch (plus strand).

The DNA sequencing reads were assembled into 12,122 multiple contigs with a minimum size more than 400 bp each. They were mapped to all cpDNA sequences available in the NCBI GenBank database including the complete chloroplast genome sequence of *B. platyfylla* (accession number NC_039994.1), and it was found that one of them, a single 160,523-bp-long contig represented a complete plastome with a high mean coverage of 207 reads per nucleotide site. It was fully annotated and deposited to the NCBI GenBank database under accession number MG966529.2. The most common nucleotides in this plastome of curly birch were A (31.5%) and T (32.5%), followed by C (18.3%) and G (17.7%). Similar nucleotide composition was observed in plastomes of other Betulaceae species (Gryta et al., 2017; Hu et al., 2017; Li et al., 2018; Wang et al., 2018). GC-rich regions were more unevenly distributed and more clustered than AT-rich regions (Figure 1).

The annotation of the curly birch plastome showed that the GC-rich regions represent mostly regions in IR1 and IR2 containing genes of rRNAs (5S, 4.5S, 23S, and 16S) and tRNAs (*trn*V, *trn*I, and *trn*A). The AT-rich regions were located mostly in the large (LSC) and small (SSC) single copies, IR1 and IR2 containing protein-coding sequences, tRNA genes, as well as intergenic spacers. The most GC-rich were rRNA (55.21%) and tRNA (53.18%) genes. The least GC-rich were protein-coding genes (37.02%), which was also noted in other studies of Betulaceae species (Hu et al., 2017; Li et al., 2018; Wang et al., 2018).

The analysis of the curly birch plastome revealed 384 microsatellite loci (Table 1). The largest number of loci was with mononucleotide motifs A and T (94% of all loci with mononucleotide motifs and 92% of all loci). However, the threshold of at least seven repeats for the loci with mononucleotide motifs could not be strict enough, and not all these loci could be real microsatellites. For example, the number of loci with seven repeats of A was 94, while with 12 repeats, only 5. These loci certainly need further verification using population samples.

**TABLE 1:** Microsatellite loci in the curly birch plastome.

Motif	Number of loci with 5–16 repeats per locus												Total number of loci
	5	6	7	8	9	10	11	12	13	14	15	16	
A	*	*	94	30	24	13	5	5	–	–	–	1	172
C	*	*	10	3	1	1	–	–	–	–	–	–	15
G	*	*	6	1	1	–	–	–	–	–	–	–	8
T	*	*	78	45	30	22	3	3	1	–	–	–	182
AT/TA	*	2	2	1	–	–	–	–	–	–	–	–	5
ATT/TTA	1	–	–	–	–	–	–	–	–	–	–	–	1
TAA/AAT	1	–	–	–	–	–	–	–	–	–	–	–	1

*Below threshold used in the search for microsatellite loci.

Microsatellite loci with di- and trinucleotide motifs represented 8% of all SRs and contained motifs only with A and T nucleotides, although the total frequency of GC and CG pairs was 5.8% in the entire plastome of curly birch.

**FIGURE 2:**
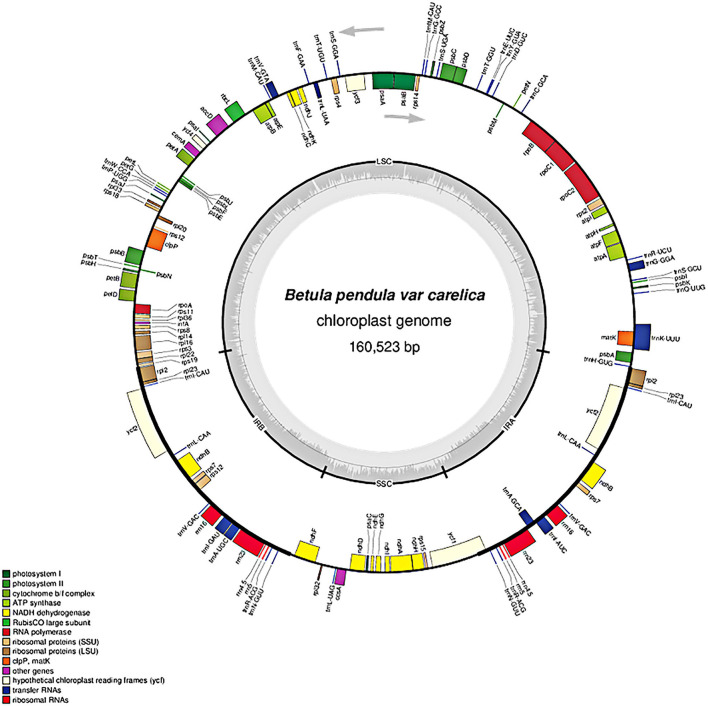
Chloroplast genome map of *Betula pendula* var. *carelica*. Arrows indicate the transcription direction. The gray inner circle displays the GC content that corresponds to the outer circle gene map. Genes belonging to different functional groups are shown in different colors.

In total, 127 coding loci representing various functional systems were identified in the plastome of curly birch (Table 2): 82 genes encoded various types of structural and catalytic proteins, 37 encoded tRNAs, and –eight encoded chloroplast rRNAs. It should be noted that out of 127 annotated loci, 95 were represented by single copies and 16 (*rpl2*, *rpl23*, *trn*I, *ycf2*, *trn*L, *ndhB*, *rps7*, *trn*V, *trn*A, *trn*I, *rrn16S*, *rrn23S*, *rrn4*, *rrn5S*, *trn*RS, and *trn*N) by two copies each. The double number of these 16 loci was due to their location in the duplicated region of the plastome—in the IR1 and IR2. The location of genes in the curly birch plastome is presented in Figure 2. It was similar to the plastomes described in other species of Betulaceae (Gryta et al., 2017; Hu et al., 2017; Li et al., 2018; Wang et al., 2018).

**TABLE 2:** Genes annotated in the curly birch plastome.

Gene	Product	Function
*ndhA*, *ndhB*, *ndhC, ndhD, ndhE, ndhF, ndhG, ndhH, ndhI, ndhJ*, and *ndhK*	Subunits of NADP dehydrogenase	Light stage of photosynthesis
*rpoA, rpoC2, rpoC1*, and *rpoB*	Polymerase subunits RNA	Gene transcription
*rrn4,5S, rrrn5S, rrrn16S*, and *rrrn23S*	Ribosomal RNA	Protein biosynthesis
*trn*A, *trn*H, *trn*K, *trn*Q, *trn*S, *trn*G, *trn*R, *trn*C, *trn*D, *trn*Y, *trn*E, *trn*T, *trn*M, *trn*L, *trn*F, *trn*V, *trn*W, *trn*P, *trn*I, and *trn*N	Transfer RNA	Protein biosynthesis
*petA, petB, petD, petG, petL*, and *petN*	Cytochromes	Light stage of photosynthesis
*rpl2, rpl14, rpl16, rpl20, rpl22, rpl32, rpl33*, and *rpl36*	Structural proteins of the large ribosome subunit	Protein biosynthesis
*rps2, rps3, rps4, rps7, rps8, rps11, rps12, rps14, rps15, rps16, rps18*, and *rps19*	Structural proteins of the minor ribosome subunit	Protein biosynthesis
*psaA, psaB, psaC, psaI*, and *psaJ*	Structural proteinsphotosystems 1	Light stage of photosynthesis
*psb*A, *psb*B, *psb*C, *psb*D, *psb*E, *psb*F, *psb*H, *psb*I, *psb*J, *psb*K, *psb*L, *psb*M, *psb*N, *psb*T, and *psb*Z	Structural proteinsphotosystems 2	Light stage of photosynthesis
*atpA, atpB, atpE, atpF, atpH*, and *atpI*	Subunits ATP synthases	ATP biosynthesis
*rbcL*	Large subunit of ribulose-1,5-bisphosphate carboxylase	Dark stage of photosynthesis
*ccsA*	Hem incorporating protein	Cytochrome C biogenesis
*matK*	Maturase	Splicing mRNA
*cemA*	Structural membrane protein chloroplast	Membrane transport
*clpP*	Proteinasa	Protein catabolism
*accD*	Acetyl-CoA carboxylase	Fatty acid biosynthesis
*ycf1, ycf2, ycf3*, and *ycf4*	Open reading frames	Undetermined

Most loci (106) consisted of a single exon and contained no introns, 18 genes (10 protein- and eight tRNA-coding) contained one intron, and three protein-coding genes contained two introns. The structural organization of the *rps12* gene was complex with three exons separated by long introns. Its exon 1 was located in the LSC, while exons 2 and 3 were located in the IR1 and IR2. The introns 1 and 2 were about 28,000 and 61,000 bp long, respectively. The considerable distance of exons from each other indicates the presence of trans-splicing during processing of mRNA for the S12 ribosomal protein.

The total number of the tRNA loci identified in the plastome of curly birch was 37, of which 23 types were represented by single copies and seven were duplicated. The isoacceptor tRNAs were identified for the following amino acids: leucine (three isoacceptor tRNA genes), isoleucine (two), serine (three), arginine (two), valine (two), glycine (two), threonine (two), and methionine (two), including formylmethionine.

The analysis of tRNA genes showed that the size of the loci varied from 71 bp (glycine tRNA-UCC) up to 93 bp (serine tRNA-UGA), with 74 bp on average. The difference in the gene’s length was also observed for the isoacceptor tRNA genes. Similar to the *rps12* gene, several tRNA genes (*trn*A-UGC, *trn*I-GAU, *trn*K-UUU, *trn*G-GCC, *trn*L-UAA, and *trn*V-UAC) contained exons located relatively far apart from each other, from 368 to 2,523 bp. The current results indicate either trans-splicing processes during tRNA maturation or functional inactivity of these genes (Hiratsuka et al., 1989; Randau et al., 2005).

Analysis of the distribution of codons in coding sequences showed that 84 loci included over 26.3 thousand triplets. Among all types of encoded amino acids, the most common were the ones characterized by the highest specific gravity: leucine (10.52%), isoleucine (8.96%), and serine (7.47%). The cysteine codons were the least frequent (1.17%). The preference for using A and T bases in the third position of the codon was obvious, which is a common feature in the structural organization of angiosperm cpDNA (Olmstead and Palmer, 1994).

Regarding starting codons in coding sequences, along with a standard ATG start codon in most reading frames, some genes were characterized by alternative codons such as GTG in the *rps19* and *psb*C genes and ACG in *ndhD*. It should be noted that these genes are not functionally associated. They are involved in different physiological processes—translation, photosynthesis, and respiration, respectively (Li et al., 2018).

**FIGURE 3:**
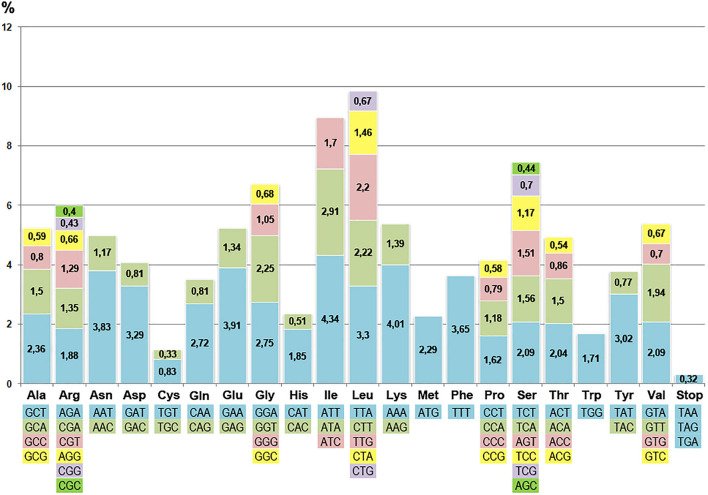
Frequency of codons of various types in the coding sequences of curly birch plastome.

The plastome does not contain all possible tRNA genes representing complementary all possible codons. There is a codon usage difference between chloroplast and nuclear genomes. It was found that the distribution of codons of the “chloroplast” and “nuclear” types had a pronounced cluster-dispersed structure. The ratio of the “chloroplast” and “nuclear” type codons for different genes differed significantly. For instance, it was significant 2:1 for the *rps15* gene but only slightly biased for *psb*C (1:1.02) and *matK* (1:1.3). The codon distribution in the curly birch plastome (Figure 3) did not correlate significantly with the frequency of respective tRNA genes. The correlation coefficient was less than 0.3.

### Comparative Analysis of the Plastome Structure and Phylogenetic Analysis

The average nucleotide difference between the curly birch plastome tRNA genes was 0.80, which shows a low similarity between nucleotide sequences of these loci. However, the level of differences between isoacceptor tRNAs did not exceed 0.15. At the same time, a comparative analysis of orthologous tRNA genes in the plastomes of various plants and in prokaryotic nucleoids of cyanobacteria showed a high degree of similarity, which indicates the absence of significant evolutionary transformations in the amino acid transport system (Biniszkiewicz et al., 1994; Paquin et al., 1997). Slight differences identified between orthologous tRNA genes were associated with single nucleotide substitutions and indels. There was no significant homology in the primary structure of tRNA with fungi and animals (Goodenbour and Pan, 2006). Thus, the results present an additional confirmation of the accepted hypothesis of the origin of higher plant chloroplasts from ancient cyanobacteria (McFadden, 2001).

A comparative analysis of chloroplast genes in various plant species at different taxonomic levels (species, genus, and family, etc.) showed that high conservatism in the structural and functional organization of the plastomes is observed among angiosperms, although the level of differences correlates directly with the taxonomic level (Sugiura, 1992). The small genetic distances detected between cpDNA sequences of curly birch and silver birch (mean D = 0.0004) are in agreement with the taxonomic status of curly birch as a variety (*B. pendula* var. *carelica*) of silver birch (*B. pendula*), respectively (Supplementary Figure 1). It should be noted that the revealed nucleotide differences were located only in non-coding regions (Supplementary Figure 1). The differences between curly birch and other species in the genus *Betula* were associated with both non-coding and coding sequences (*ndhF*, *ycf1*, *matK*, and *atpI* genes) (Supplementary Figures 1, 2).

**FIGURE 4:**
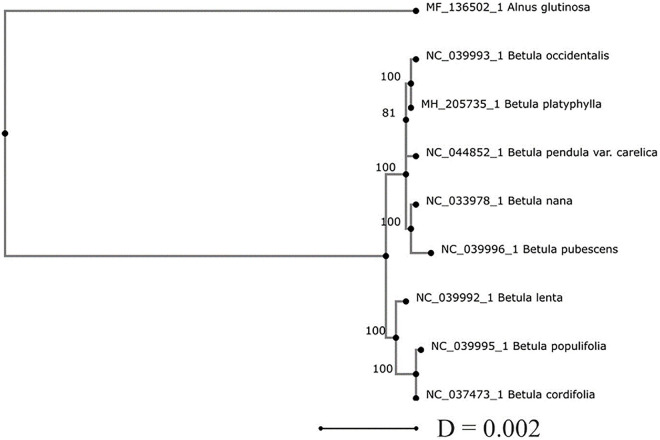
The consensus neighbor-joining (NJ) phylogenetic tree of eight *Betula* species and the out-group *Alnus glutinosa* species based on their pairwise Jukes–Cantor (Jukes and Cantor, 1969) genetic distances (D) computed for the whole plastome sequences. The numbers at the cluster nodes are percentages of trees in which the associated taxa clustered together based on 1,000 bootstraps.

Phylogenetic analysis of the genus *Betula* using cpDNA sequences available at the NCBI GenBank showed that the level of genetic differences between *Betula* species is low and does not allow their reliable taxonomic classification (Figure 4). Among eight birch species presented in the NJ tree, six species *B. pendula* var. *carelica*, *B. pubescens*, *B. platyphylla*, *B. populifolia*, *B. occidentalis*, and *B. cordifolia* belong to the subgenus *Betula* (De Jong, 1993), *B. nana* belongs to the subgenus *Chamaebetula*, and *B. lenta* to the subgenus *Betulenta*.

The species of subgenus *Chamaebetula* and *Betulenta* occupied an intermediate position between species of subgenus *Betula*. These data may indicate that the range of geographic variability detected within each of the species may correlate with the level of interspecific genetic differences. This phylogenetic uncertainty may be due to the recent origin of birch species and a high level of introgressive hybridization and the corresponding gene flow between them.

The level of genetic similarity within the *Betulaceae* family was about 99% based on alignments of *Betulaceae* species using dot plots and data available in the NCBI GenBank database (Supplementary Figures 2, 3). Differences were mainly due to single nucleotide substitutions rather than indels, and no major rearrangements or inversions of nucleotide sequences were detected. Most variation was observed in non-coding intergenic regions *trn*R-TCT*–atpA*, *trn*E-TTC*–trn*T-GGT, *psb*E*–petL*, and *rpl32–trn*L-TAG. Among genes, the *ycf1* gene was the most polymorphic.

The size of IR1 and IR2 varied slightly (<2%) and was about 26,000 bp on average in the compared angiosperms. The structure of inverted repeats was characterized by several features in curly birch: the *rps19* gene was located in the LSC in European beech (*Fagus sylvatica*) but stretched partly over the IR2 in curly birch. The *rpl2* gene was completely located inside the IR2 in species other than birches, but in *Betula* spp., it was outside of the IR2, near its left border. The ψ*ycf1* pseudogene was present in all angiosperm plastomes. It is located inside IR2 in all species except coconut palm (*Cocos nucifera*), in which it continued into the SSC for several nucleotides. The distance between the ψ*ycf1* and *ndhF* genes is the longest in birches, while the shortest is in European boxwood (*Buxus sempervirens*). In all studied angiosperms, the *ycf1* gene is located across an inverted repeat (IR1) border, but mostly within IR1 in birches. The pseudogene ψ*rps19* was found only in birches and is located in the IR1, next to the border with the LSC. The *trn*H-GTG gene was also located in the LSC, 7–47 bp apart from the IR1-LSC border.

In total, 254 cpSSR markers (Supplementary Table 1) and 86 EST-cpSSR markers (Supplementary Table 2) were discovered, and their prospective PCR primers were developed that can be potentially used in genetic studies of curly birch after verification.

The chloroplast markers are very efficient in phylogenetic studies because they are haploid, which unlike nuclear markers makes it easier to genetically compare diploid species with polyploid ones (Wang et al., 2018; Lee et al., 2020). The chloroplast genome has a strictly maternal inheritance in most angiosperms including birch (Sears, 1980; Reboud and Zeyl, 1994), and therefore, it is distributed by seeds that have relatively limited distribution compared to pollen, which makes chloroplast markers very efficient markers also to study spatial genetic structure, post-glacial colonization, and phylogeography (Palmé et al., 2003; Tsuda and Ide, 2010). They can be used to study the direction of introgressive hybridization, which is very common in birch species, by comparing phylogeny based on maternally inherited chloroplast markers with phylogeny based on the bi-parentally inherited nuclear markers. The chloroplast genomes are much more abandoned than nuclear ones and are better preserved in the ancient and environmental DNA that is widely used now for different analyses such as sedimentary analyses that allow inferring the flora structure and related climate in the past (Meucci et al., 2021).

The haplotype analysis data of the of cpDNA of curly birch can be used to assess forest seed areas within which the transfer of seed and planting material is carried out. In addition, cpDNA markers are used to study mating systems in seed orchards and verify their origin.

The studied curly birch plastome is not significantly different from plastomes in other angiosperms of woody plant species in its structural and functional organization (Sugiura, 1992; Gryta et al., 2017; Hu et al., 2017; Li et al., 2018; Wang et al., 2018; Supplementary Figure 1). Moreover, our preliminary study of haplotype diversity based on the cpSSR markers demonstrated also that natural populations of silver and curly birches have a similar set of chloroplast haplotypes, which shows their close relationships and origin of curly birch from silver birch (unpublished data). It confirms conclusions based on the hybridological and population analysis that patterned wood of curly birch is an inherited Mendelian trait controlled by nuclear genes. This is also confirmed by the population genetic structure analysis of the curly birch natural stands. It is well-known that populations of curly birch are scattered around the world and spatially isolated from one another, which significantly limits the gene flow between them (Butorina, 1993; Koski and Rousi, 2005; Vetchinnikova, 2005; Vetchinnikova et al., 2013; Isakov et al., 2017; Moshchenskaya et al., 2017). In our preliminary analysis based on the cpDNA microsatellite markers, we observed that curly birches in Belarus were more similar to silver birches in Belarus than to curly birches in Ukraine and *vice versa*—curly birches in Ukraine were more similar to silver birches in Ukraine than to curly birches in Belarus (unpublished data). It demonstrates that cpDNA structure has no significant effect on the anatomical traits of wood. However, it is still unclear whether this curly birch-specific decorative texture of the wood is controlled by the same gene(s) in different populations located far from each other. Further search for genetic markers associated with the expression of this curly-grain pattern trait is needed.

Despite the significant number of published studies devoted to the analysis of plastomes in higher plants, interaction of nucleus and chloroplast genes and their coevolution is still not fully understood (Zhang et al., 2015; Weng et al., 2016; Ferreira de Carvalho et al., 2019). From our point of view, the nucleotide composition bias in plastids is of particular interest. As indicated earlier, the GC content of LSC and SSC differed significantly from the rRNA regions in inverted repeats. Despite the bias in cpDNA toward GC bases in general, some protein-coding regions are biased toward A and T. The same is observed in cpDNA in phylogenetically different plants. It is also a characteristic of most of the described modern cyanobacteria (Ling et al., 2015; Grosjean and Westhof, 2016; Chin, 2017; Mukai et al., 2017). Unlike the rRNA genes in the plastome, the rRNA genes in mitochondrial genomes of eukaryotic species are characterized by a lower frequency of G and C bases despite their similar roles in both organelle genomes. They also have a general similarity in their distribution among the other regions of mitochondrial DNA (Newton, 1988; Small et al., 1989; Burger et al., 2003).

## Conclusion

The complete plastome sequence of curly birch (*B pendula* var. *carelica*), one of the most peculiar and rarest trees in the entire North and Central Europe, was *de novo* assembled and annotated for the first time. It was compared to plastomes in silver birch and other Betulaceae species. Comparative analysis revealed a small genetic difference between curly and silver birches, confirming their taxonomic status. Small differences between curly birch and other Betulaceae species were associated with both non-coding and coding regions. The level of genetic similarity between plastomes of species in the Betulaceae family was about 99%. We found highly variable loci that could be used potentially as polymorphic molecular genetic markers in the future population and phylogenetic studies. The polymorphisms found in this study, which distinguish *B. pendula* var. *carelica* from other Betulaceae species, could be useful for designing markers for breeding programs. The complete curly birch plastome sequence is also useful for understanding phylogenetic relationships among Betulaceae species.

## Data Availability Statement

The datasets presented in this study can be found in online repositories. The names of the repository/repositories and accession number(s) can be found in the article/Supplementary Material.

## Author Contributions

OB, VP, KK, and KS designed the research. OB, PK, LM, and SP collected and analyzed the data. OB, KK, KS, and EM wrote the manuscript. OB, VP, KK, KS, and EM discussed the results and revised the manuscript. All authors contributed to the article and approved the submitted version.

## Conflict of Interest

The authors declare that the research was conducted in the absence of any commercial or financial relationships that could be construed as a potential conflict of interest.

## Publisher’s Note

All claims expressed in this article are solely those of the authors and do not necessarily represent those of their affiliated organizations, or those of the publisher, the editors and the reviewers. Any product that may be evaluated in this article, or claim that may be made by its manufacturer, is not guaranteed or endorsed by the publisher.

## Footnotes

1
https://www.biologicscorp.com/tools/GCContent/

## Supplementary Material

Supplementary Figure 1 Multiple nucleotide sequence alignment of nine woody plant species plastomes from *Spermatophyta* (mVISTA). *B. pendula var. carelica* was used as a reference. Gray arrows above the alignment indicate the transcriptional directions of genes. Genome regions are color-coded as exon (violet) and conserved non-coding sequences (CNS) (red). A cut-off of 50% identity was used for the plots. The Y-axis indicates the percent identity between 50 and 100%.

Supplementary Figure 2 Multiple whole-plastome sequence alignment of eight birch species. Gray arrows above the alignment indicate the transcriptional directions of genes. Genome regions are color-coded.

Supplementary Figure 3 Dot plots of pairwise alignments of the *B. pendula var. carelica* (MG966529.2) complete plastome sequence vs. plastomes of eight birch species.

Supplementary Table 1 The PCR primers for cpDNA curly birch microsatellite (cpSSR) loci.

Supplementary Table 2 The PCR primers designed to amplify SSR in structurally functional genes in the curly birch cpDNA (EST-cpSSR markers).

## Abbreviations

cpDNA: chloroplast DNA
cpSSR: chloroplast SSR
EST: expressed sequence tag
LSC: large single copy
MCL: maximum composite likelihood
ML: maximum likelihood
NJ: neighbor-joining
SSC: small single copy
SSR: simple sequence repeat
WPM: Woody Plant Medium.
