# Supplementary material for: Structure and Phylogeny of the Curly Birch Chloroplast Genome
Source: Front Genet. 2021 Oct 4;12:625764. doi: 10.3389/fgene.2021.625764 (PMC8521055; doi:10.3389/fgene.2021.625764)
Supplement: Supplementary Figure 1 — Multiple nucleotide sequence alignment of nine woody plant species plastomes from Spermatophyta (mVISTA). B. pendula var. carelica was used as a reference. Gray arrows above the alignment indicate the transcriptional directions of genes. Genome regions are color-coded as exon (violet) and conserved non-coding sequences (CNS) (red). A cut-off of 50% identity was used for the plots. The Y-axis indicates the percent identity between 50 and 100%. [file Image_1.PDF]

Supplementary Material

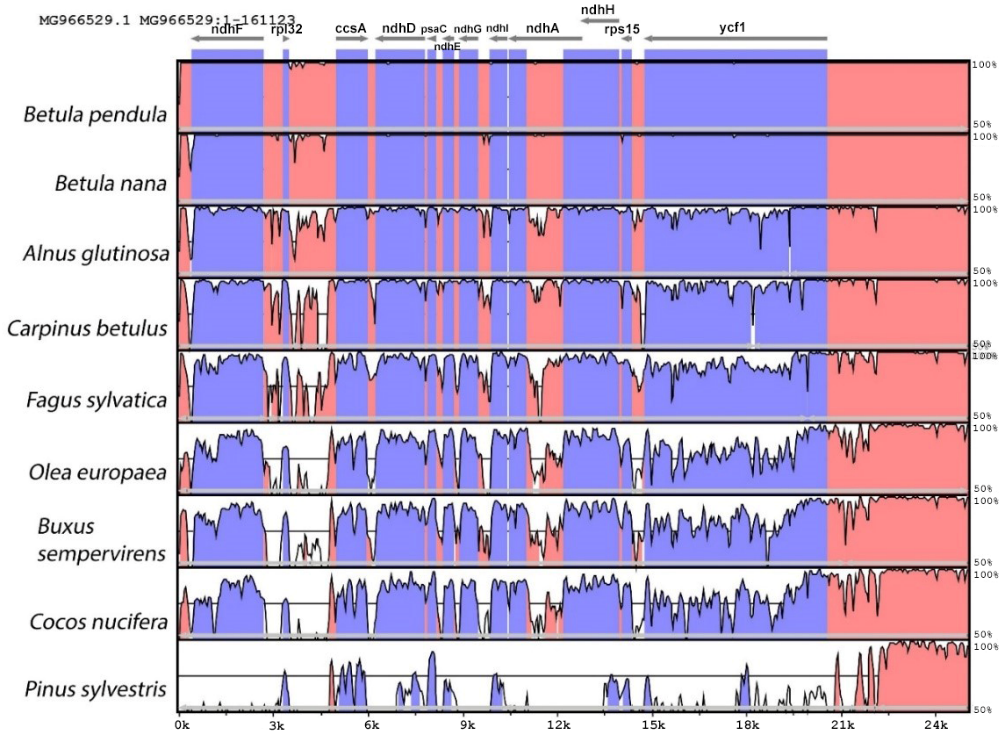

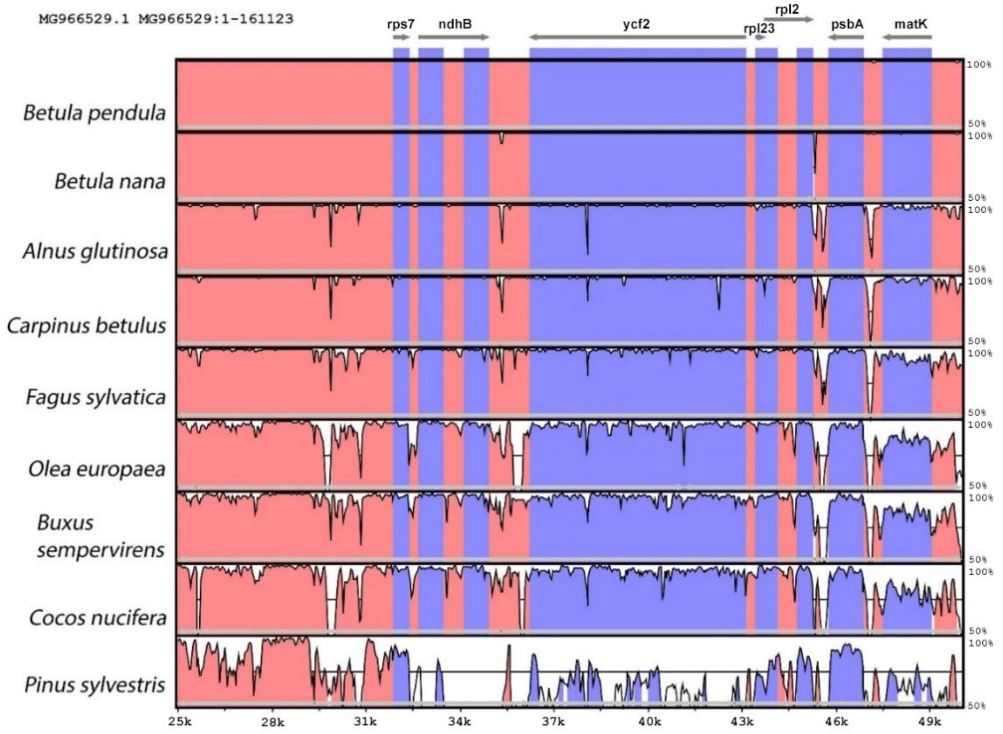

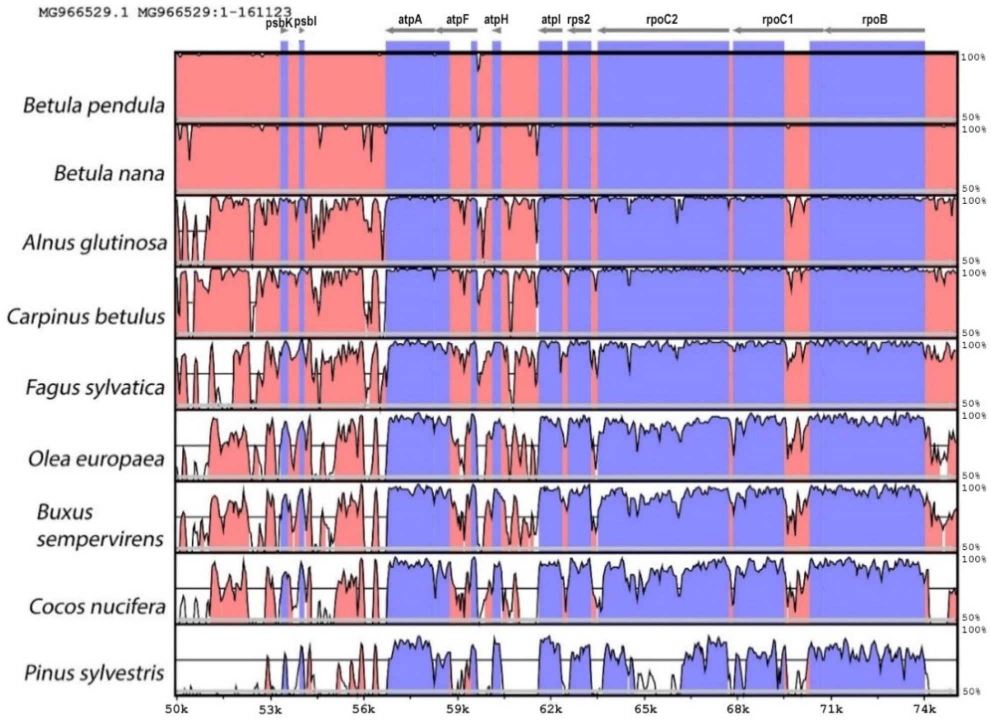
MG966529.1 MG966529:1-161123
psbK psbI
atpA
atpF
atpH
atpI
rps2
rpoC2
rpoC1
rpoB
Betula pendula
Betula nana
Alnus glutinosa
Carpinus betulus
Fagus sylvatica
Olea europaea
Buxus sempervirens
Cocos nucifera
Pinus sylvestris
100%
50%
50k
53k
56k
59k
62k
65k
68k
71k
74k

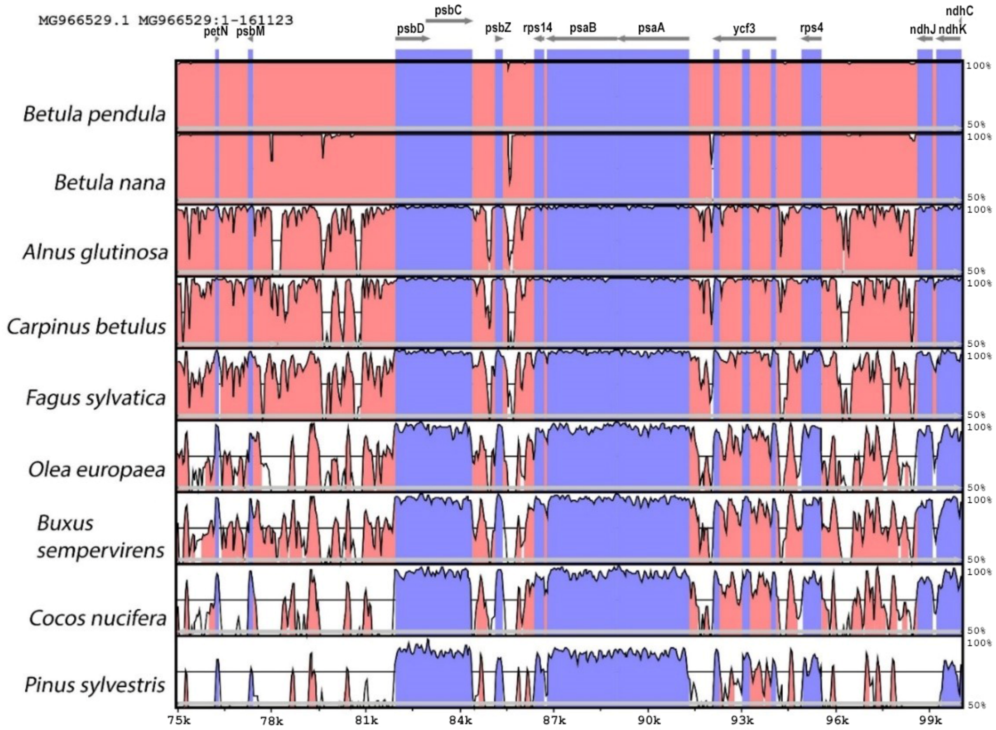
MG966529.1 MG966529:1-161123
petN
psbM
psbD
psbC
psbZ
rps14
psaB
psaA
ycf3
rps4
ndhJ
ndhK
ndhC
Betula pendula
Betula nana
Alnus glutinosa
Carpinus betulus
Fagus sylvatica
Olea europaea
Buxus sempervirens
Cocos nucifera
Pinus sylvestris
100%
50%
75k
78k
81k
84k
87k
90k
93k
96k
99k

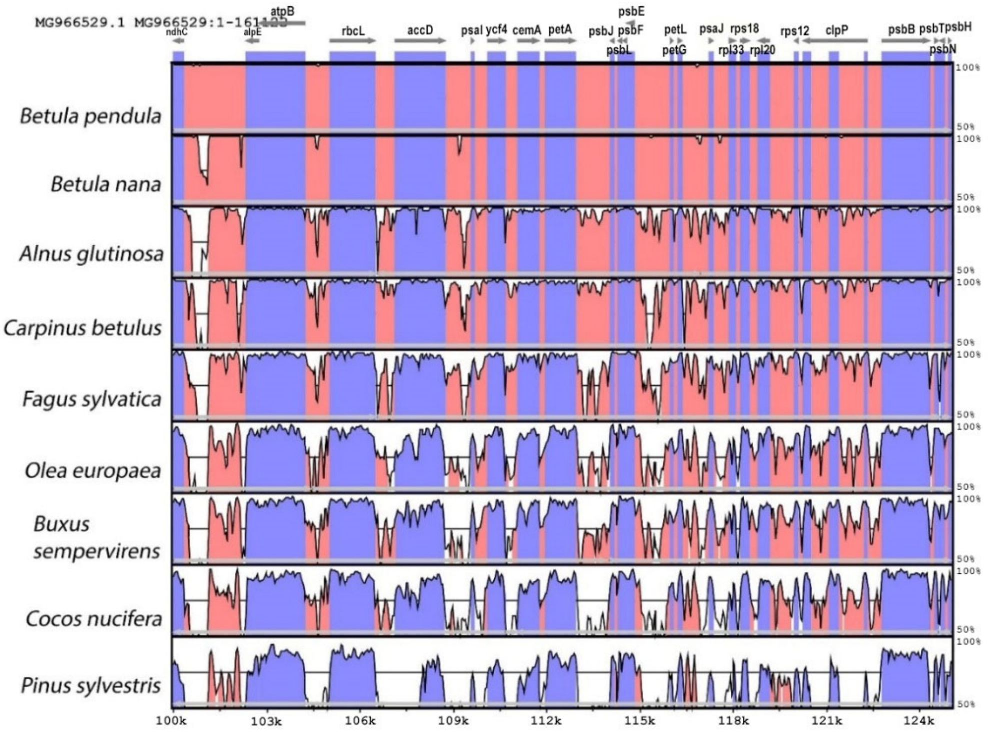
MG966529.1 MG966529:1-161123
ndhC atpE atpB rbcL accD psaI ycf4 cemA petA psbJ psbL psbE psbF petL petG psaJ rpl33 rps18 rpl20 rps12 clpP psbB psbT psbN psbH
Betula pendula
Betula nana
Alnus glutinosa
Carpinus betulus
Fagus sylvatica
Olea europaea
Buxus sempervirens
Cocos nucifera
Pinus sylvestris
100%
50%
100k 103k 106k 109k 112k 115k 118k 121k 124k

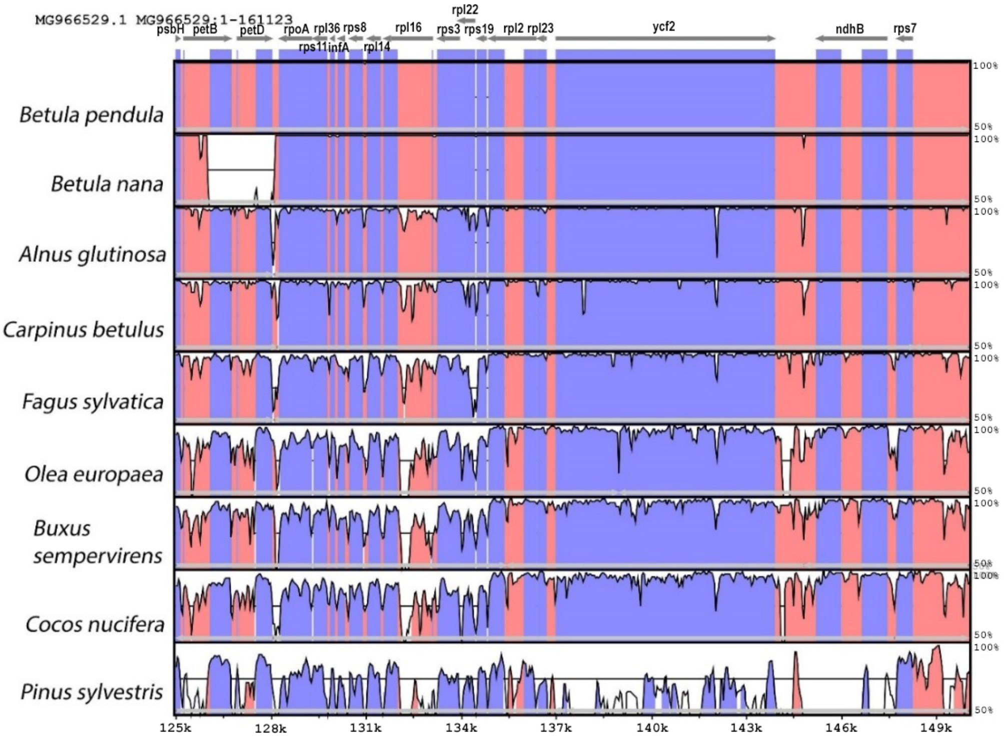
MG966529.1 MG966529:1-161123
psbH petB petD rpoA rps11 rpl36 infA rps8 rpl14 rpl16 rpl22 rps3 rps19 rpl2 rpl23 ycf2 ndhB rps7
Betula pendula
Betula nana
Alnus glutinosa
Carpinus betulus
Fagus sylvatica
Olea europaea
Buxus sempervirens
Cocos nucifera
Pinus sylvestris
100%
50%
125k 128k 131k 134k 137k 140k 143k 146k 149k

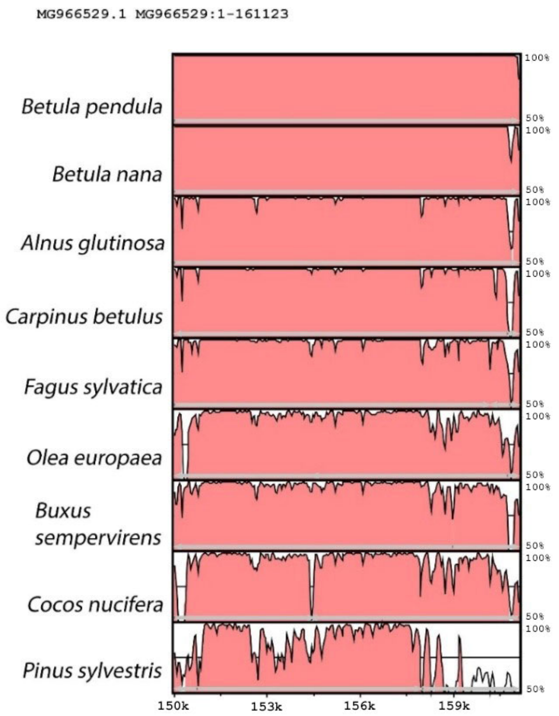

Supplementary Figure S1. Multiple nucleotide sequence alignment of nine woody plant species plastomes from *Spermatophyta* (mVISTA). *B. pendula var. carelica* was used as a reference. Grey arrows above the alignment indicate the transcriptional directions of genes. Genome regions are color-coded as exon (violet) and conserved non-coding sequences (CNS) (red). A cut-off of 50% identity was used for the plots. The Y-axis indicates the percent identity between 50 and 100%.

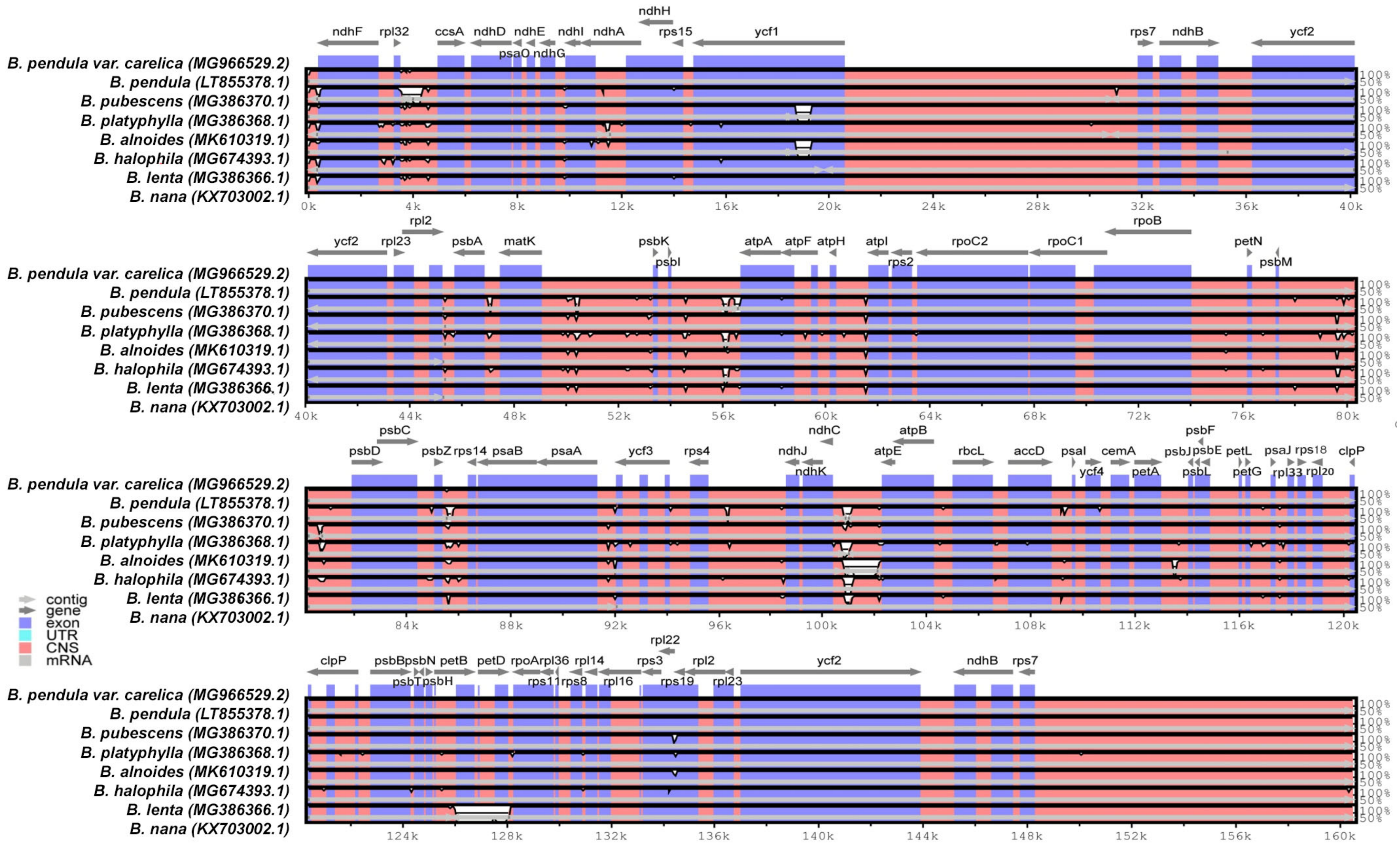

Supplementary Figure S2. Multiple whole-plastome sequence alignment of eight birch species. Grey arrows above the alignment indicate the transcriptional directions of genes. Genome regions are color-coded.

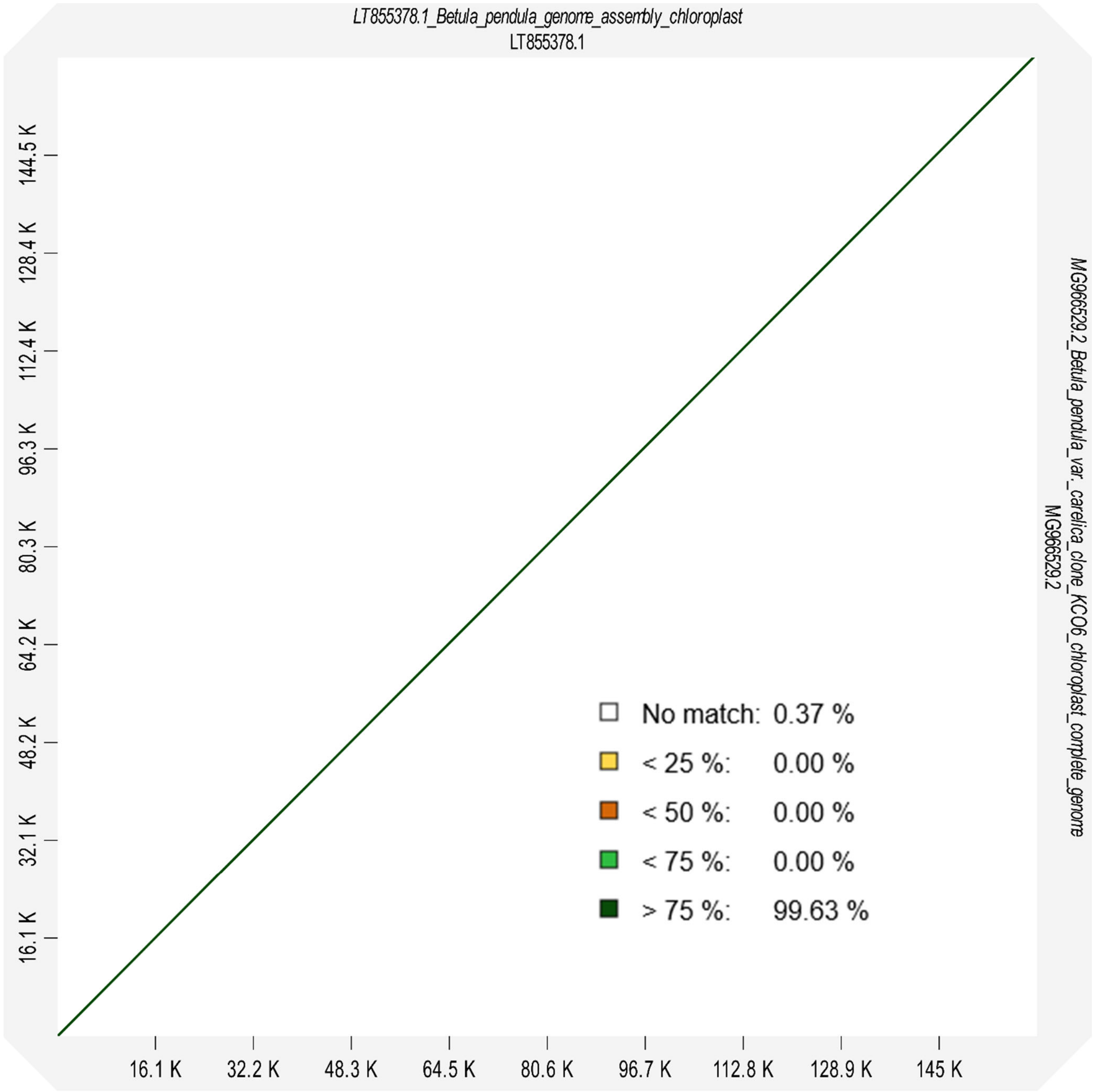
LT855378.1_Betula_pendula_genome_assembly_chloroplast
LT855378.1
MG966529.2_Betula_pendula_var._carelica_clone_KCO6_chloroplast_complete_genome
MG966529.2
144.5 K
128.4 K
112.4 K
96.3 K
80.3 K
64.2 K
48.2 K
32.1 K
16.1 K
16.1 K
32.2 K
48.3 K
64.5 K
80.6 K
96.7 K
112.8 K
128.9 K
145 K
No match: 0.37 %
< 25 %: 0.00 %
< 50 %: 0.00 %
< 75 %: 0.00 %
> 75 %: 99.63 %

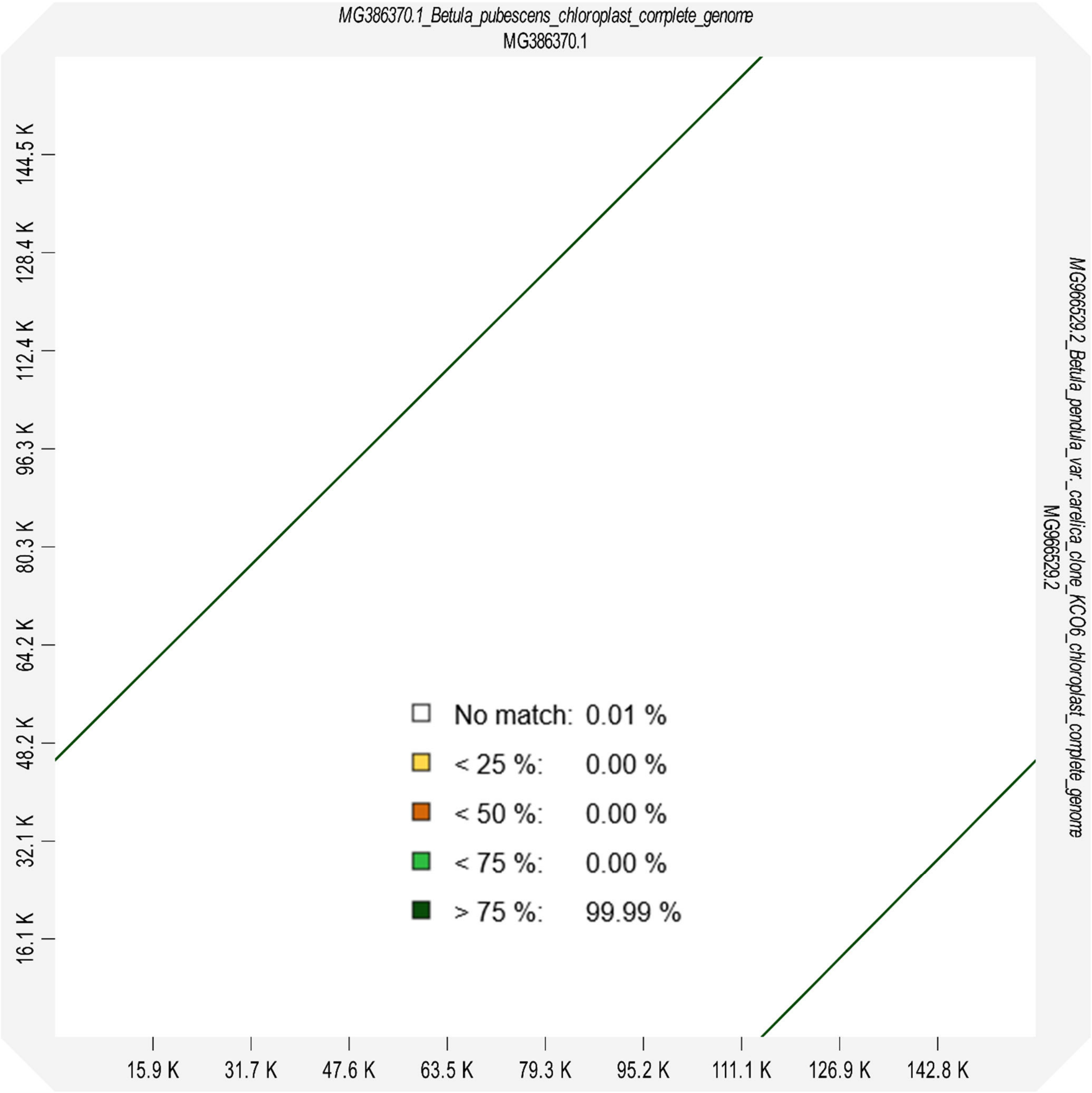
MG386370.1_Betula_pubescens_chloroplast_complete_genome
MG386370.1
MG966529.2_Betula_pendula_var._carelica_clone_KCO6_chloroplast_complete_genome
MG966529.2
144.5 K
128.4 K
112.4 K
96.3 K
80.3 K
64.2 K
48.2 K
32.1 K
16.1 K
15.9 K
31.7 K
47.6 K
63.5 K
79.3 K
95.2 K
111.1 K
126.9 K
142.8 K
No match: 0.01 %
< 25 %: 0.00 %
< 50 %: 0.00 %
< 75 %: 0.00 %
> 75 %: 99.99 %

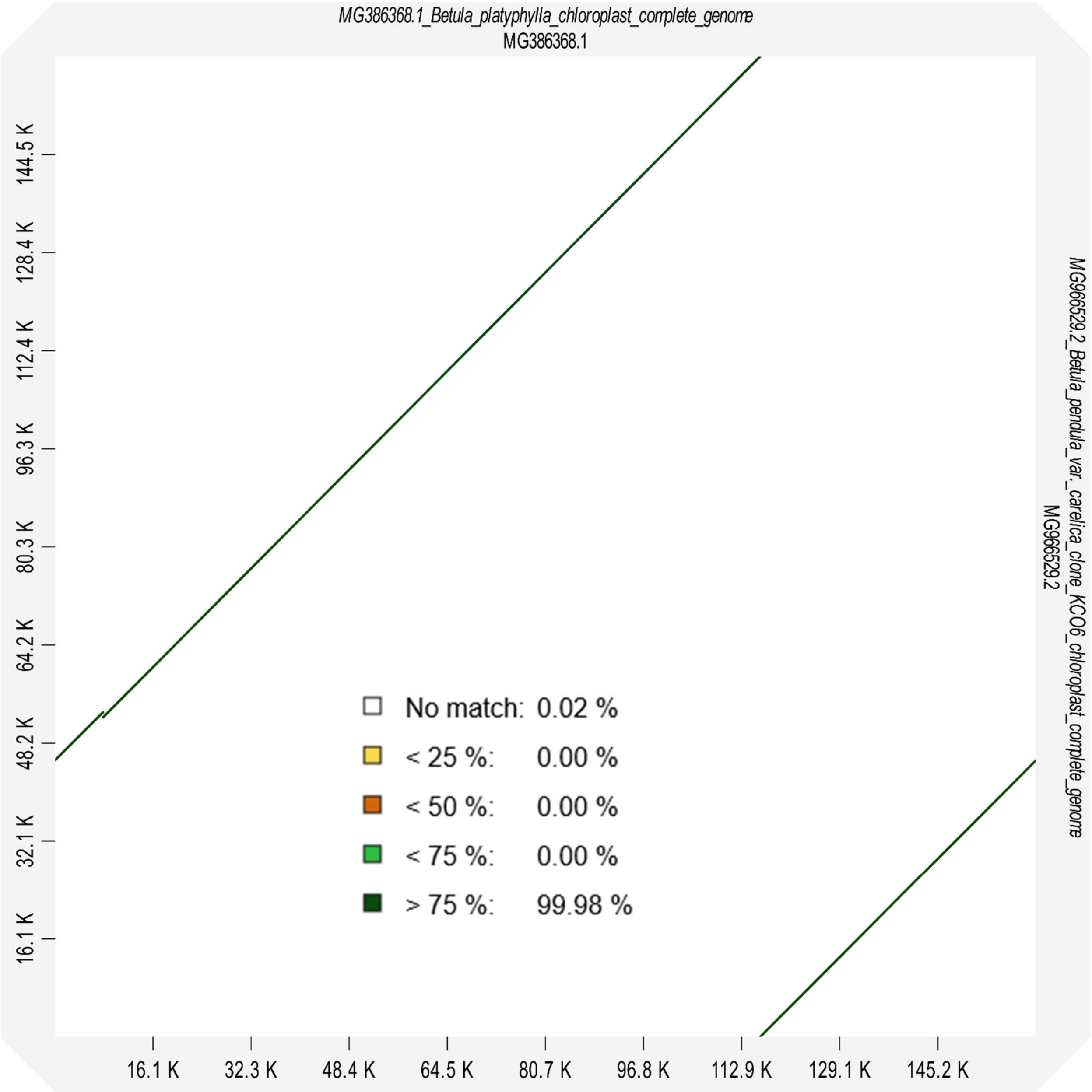

MG386368.1_Betula_platyphylla_chloroplast_complete_genome
MG386368.1
MG966529.2_Betula_pendula_var._carelica_clone_KCO6_chloroplast_complete_genome
MG966529.2
144.5 K
128.4 K
112.4 K
96.3 K
80.3 K
64.2 K
48.2 K
32.1 K
16.1 K
No match: 0.02 %
< 25 %: 0.00 %
< 50 %: 0.00 %
< 75 %: 0.00 %
> 75 %: 99.98 %
16.1 K
32.3 K
48.4 K
64.5 K
80.7 K
96.8 K
112.9 K
129.1 K
145.2 K

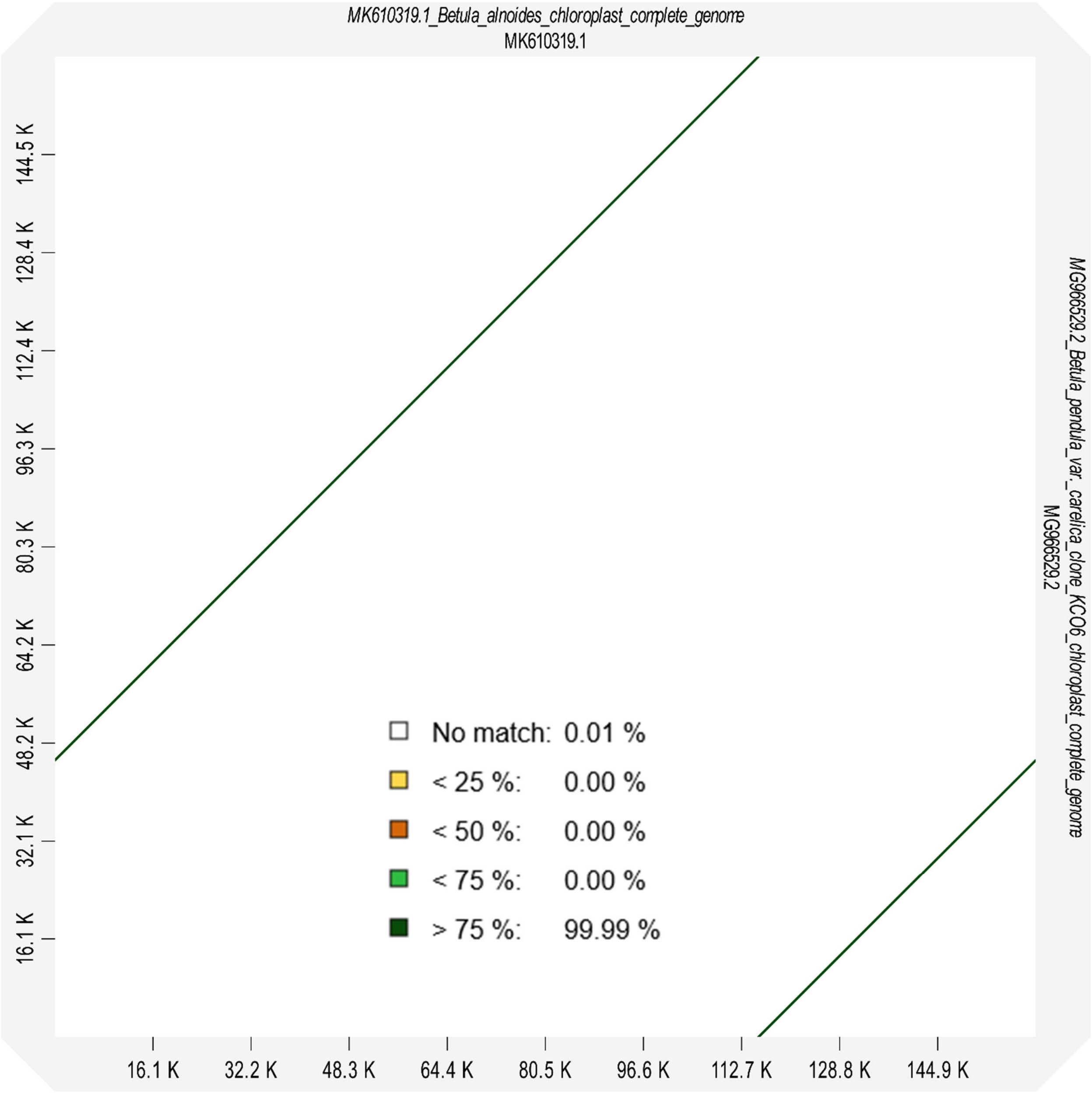
MK610319.1_Betula_alnoides_chloroplast_complete_genome
MK610319.1
MG966529.2_Betula_pendula_var._carelica_clone_KCO6_chloroplast_complete_genome
MG966529.2
144.5 K
128.4 K
112.4 K
96.3 K
80.3 K
64.2 K
48.2 K
32.1 K
16.1 K
16.1 K
32.2 K
48.3 K
64.4 K
80.5 K
96.6 K
112.7 K
128.8 K
144.9 K
No match: 0.01 %
< 25 %: 0.00 %
< 50 %: 0.00 %
< 75 %: 0.00 %
> 75 %: 99.99 %

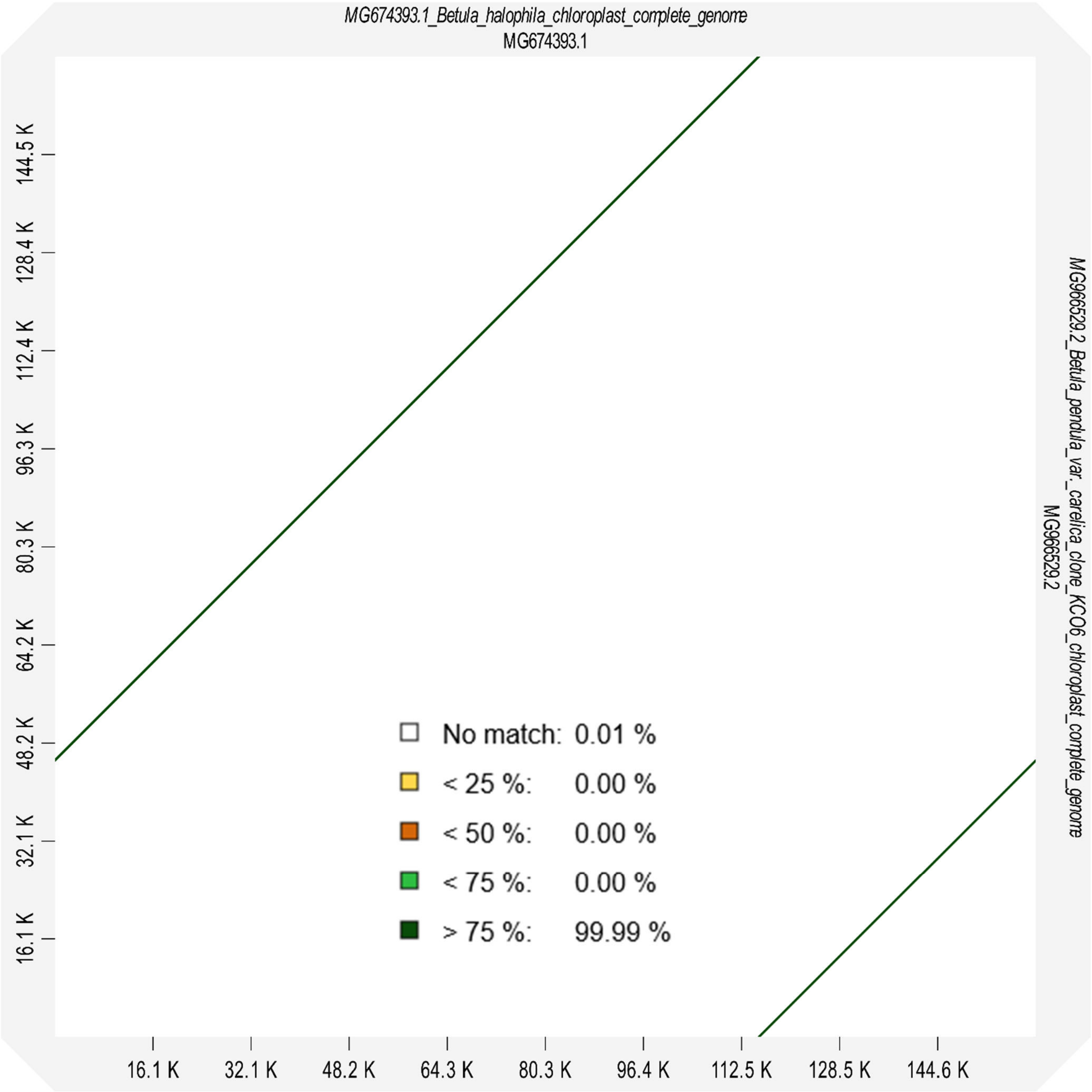
MG674393.1_Betula_halophila_chloroplast_complete_genome
MG674393.1
MG966529.2_Betula_pendula_var._carelica_clone_KCO6_chloroplast_complete_genome
MG966529.2
144.5 K
128.4 K
112.4 K
96.3 K
80.3 K
64.2 K
48.2 K
32.1 K
16.1 K
16.1 K
32.1 K
48.2 K
64.3 K
80.3 K
96.4 K
112.5 K
128.5 K
144.6 K
No match: 0.01 %
< 25 %: 0.00 %
< 50 %: 0.00 %
< 75 %: 0.00 %
> 75 %: 99.99 %

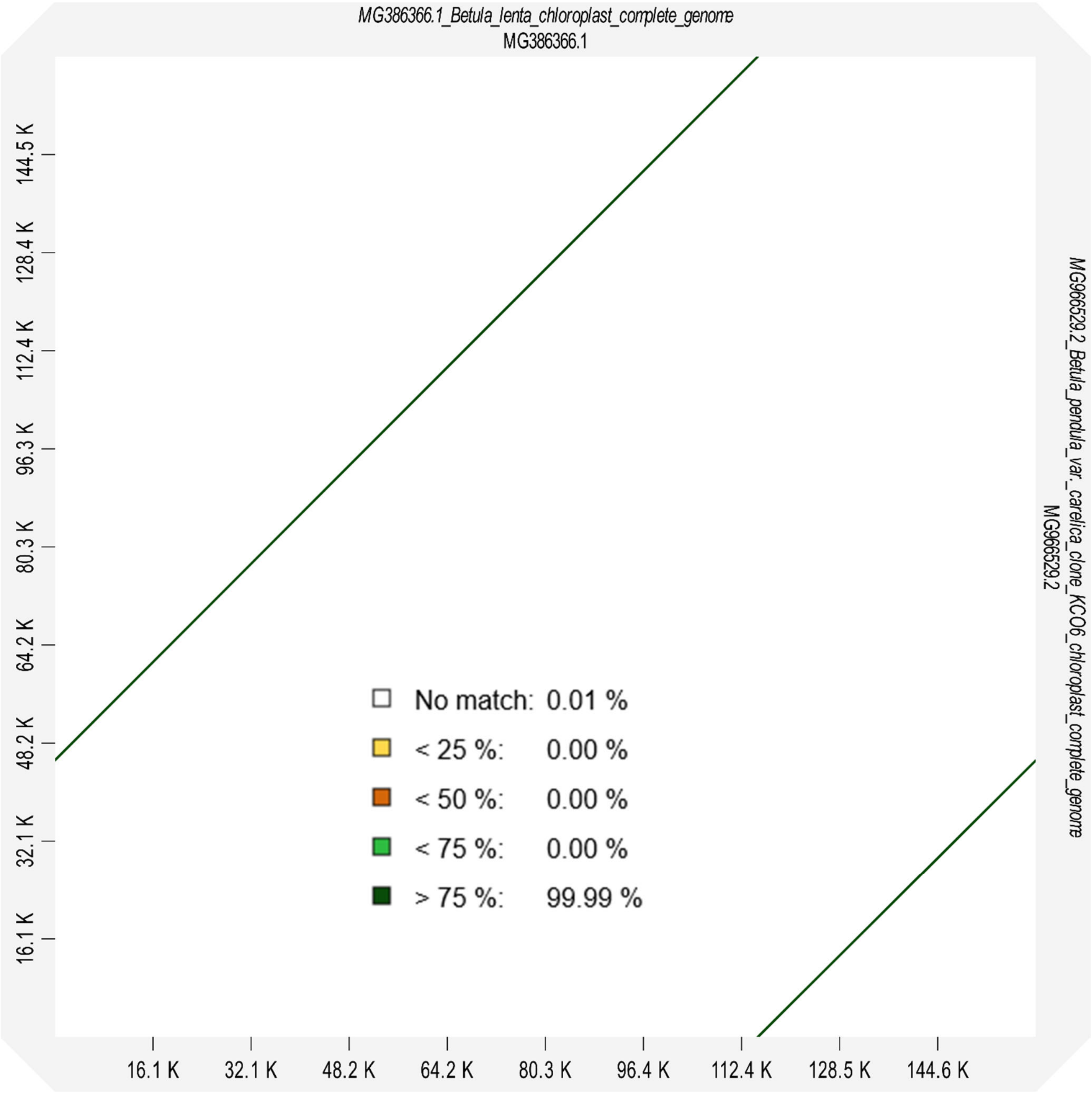
MG386366.1_Betula_lenta_chloroplast_complete_genome
MG386366.1
MG966529.2_Betula_pendula_var._carelica_clone_KCO6_chloroplast_complete_genome
MG966529.2
16.1 K
32.1 K
48.2 K
64.2 K
80.3 K
96.4 K
112.4 K
128.5 K
144.6 K
16.1 K
32.1 K
48.2 K
64.2 K
80.3 K
96.3 K
112.4 K
128.4 K
144.5 K
No match: 0.01 %
< 25 %: 0.00 %
< 50 %: 0.00 %
< 75 %: 0.00 %
> 75 %: 99.99 %

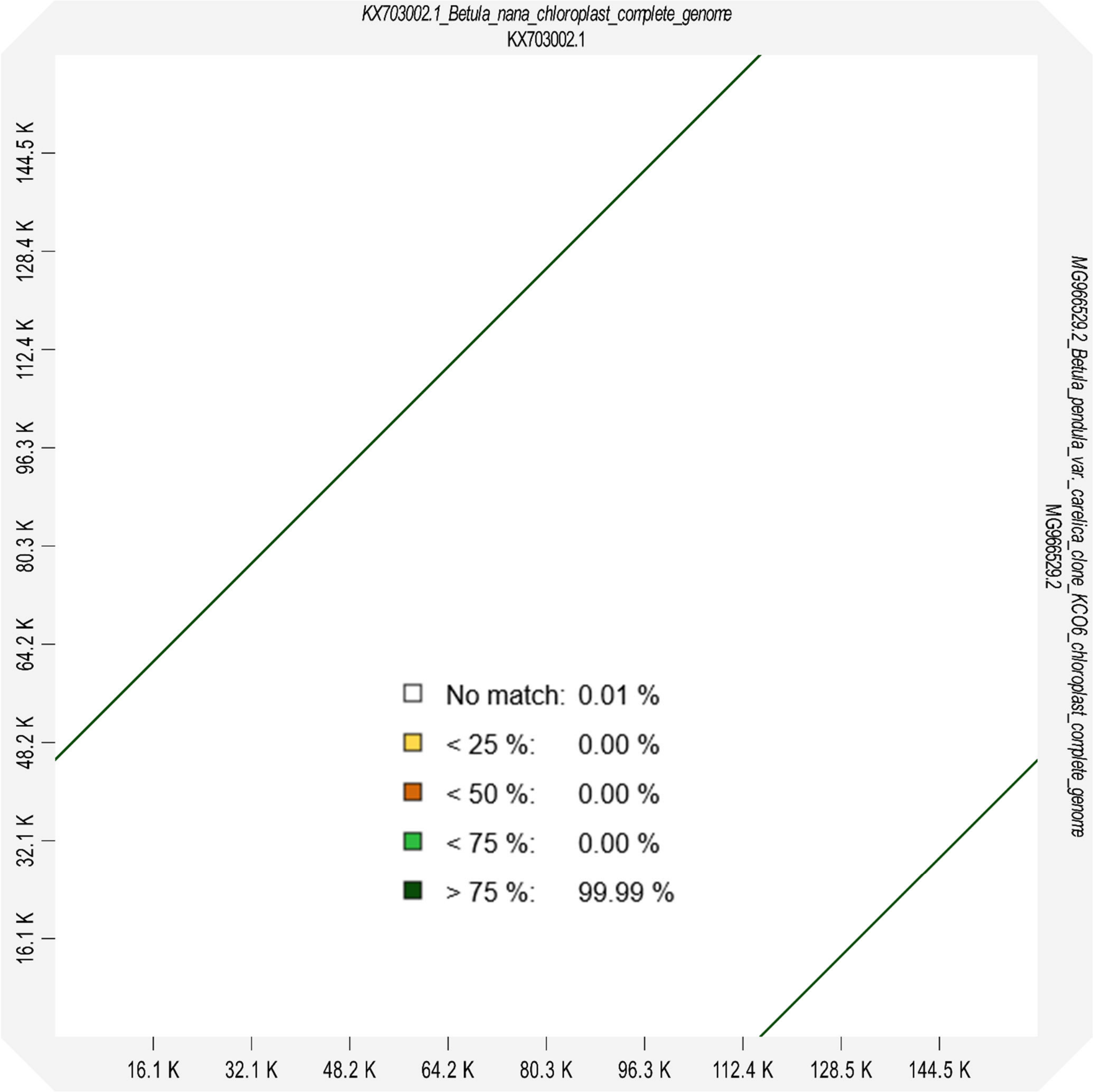

Supplementary Figure S3. Dot plots of pairwise alignments of the *B. pendula var. carelica* (MG966529.2) complete plastome sequence vs. plastomes of eight birch species.
