# Supplementary material for: Structure and Phylogeny of the Curly Birch Chloroplast Genome
Source: Front Genet. 2021 Oct 4;12:625764. doi: 10.3389/fgene.2021.625764 (PMC8521055; doi:10.3389/fgene.2021.625764)
Supplement: Supplementary file 3 [file Data_Sheet_2.docx]

Supplementary Material

| 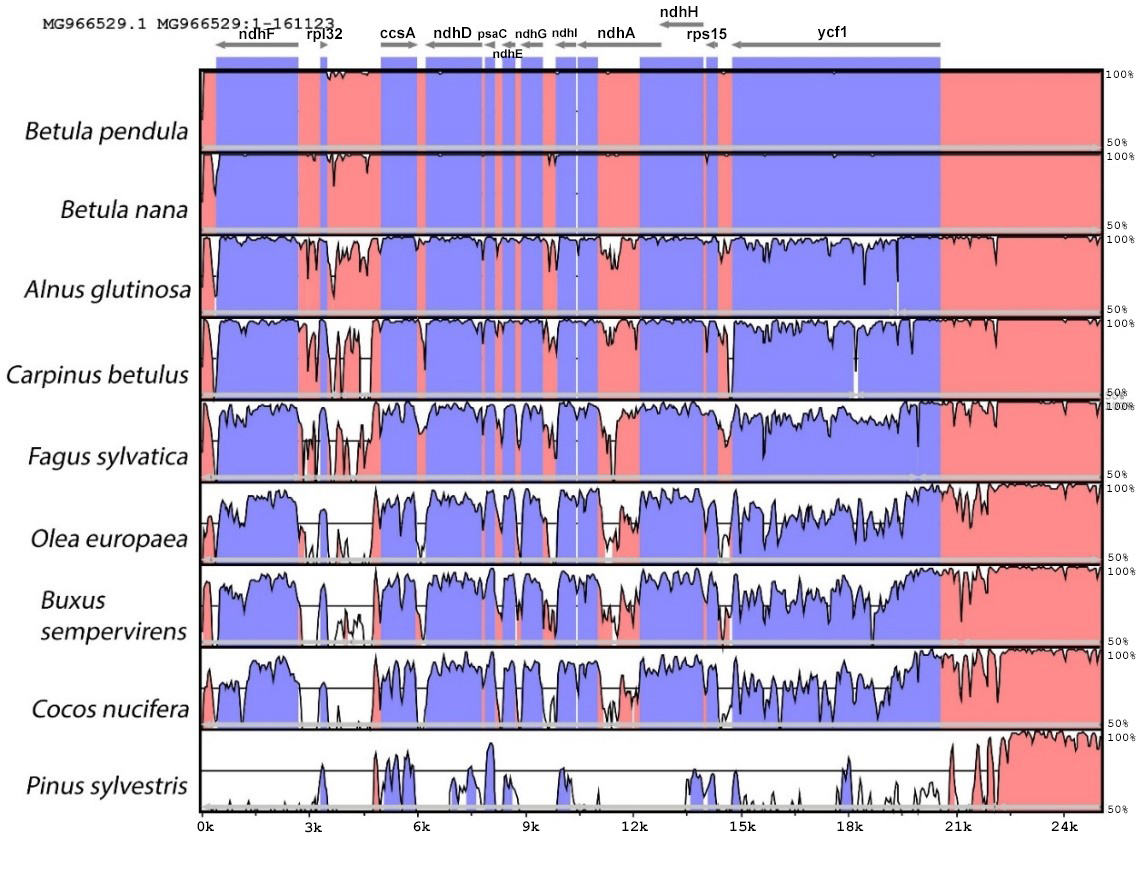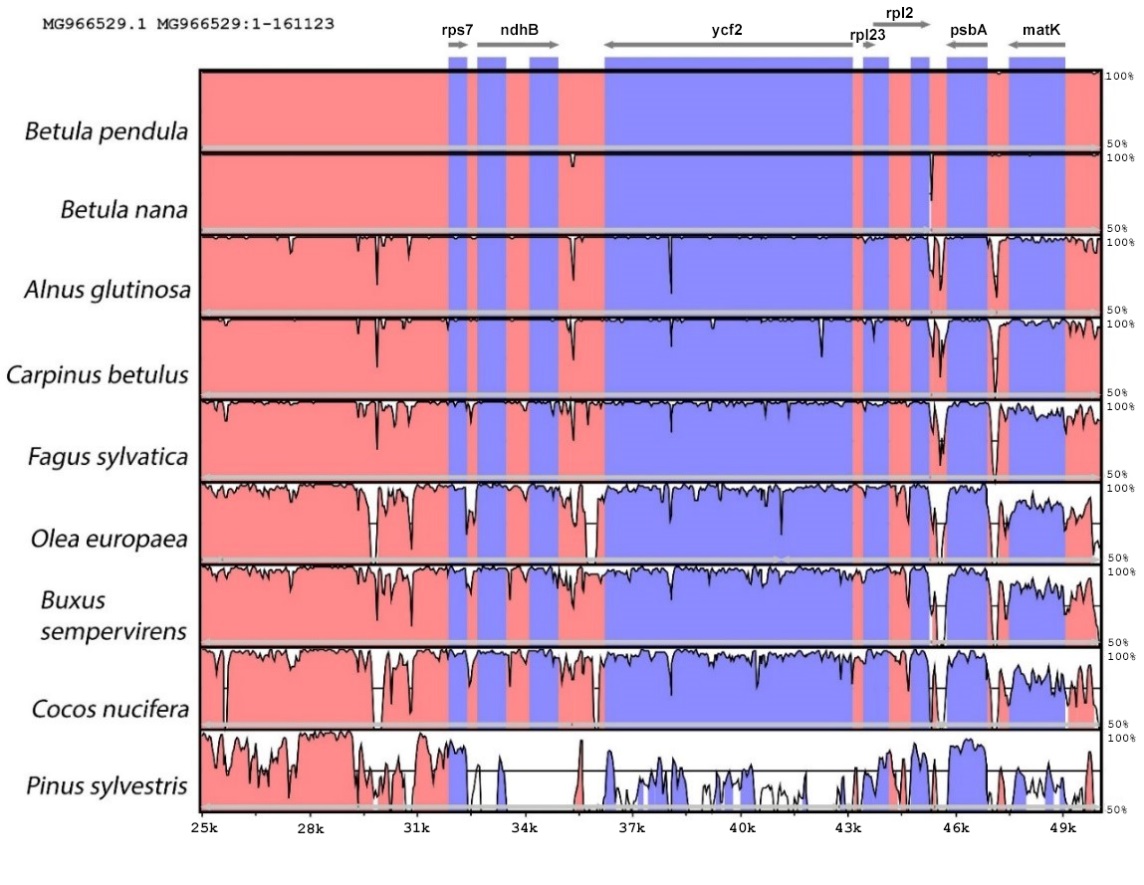 |
| --- |
| 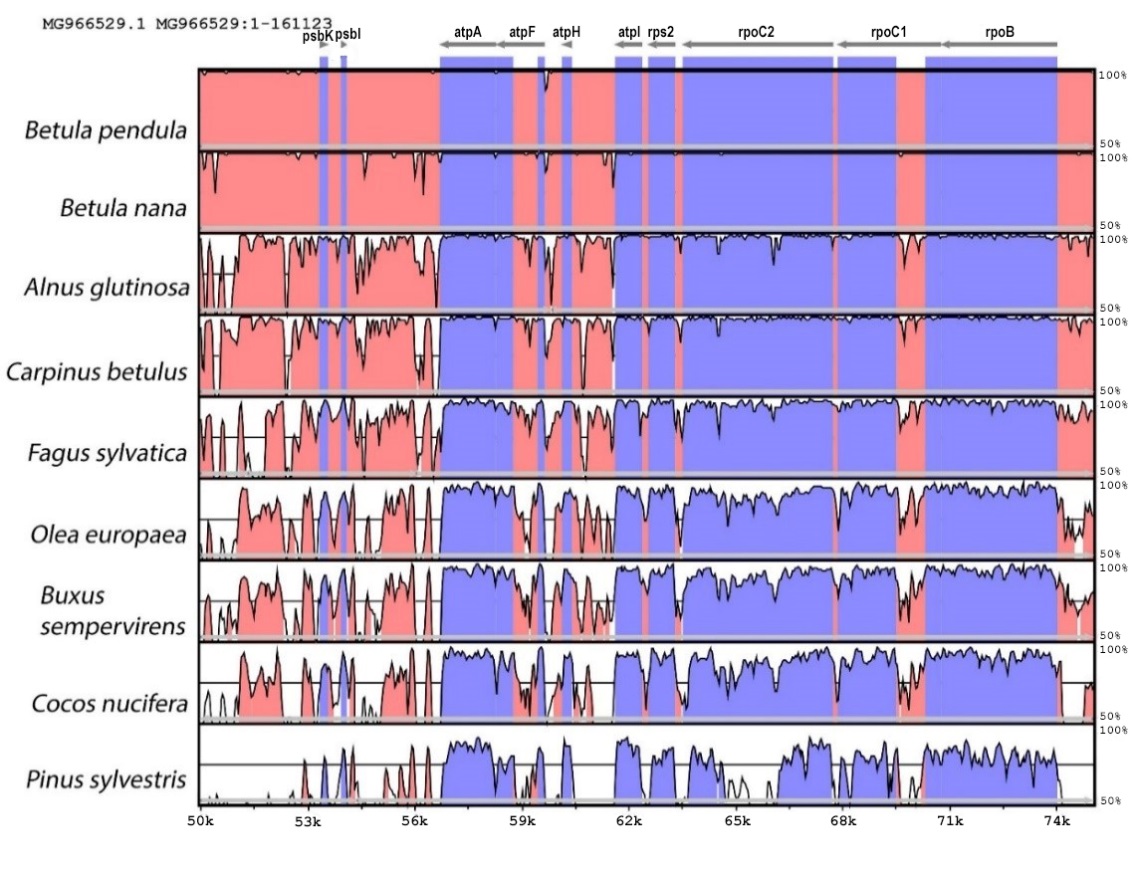 |
| 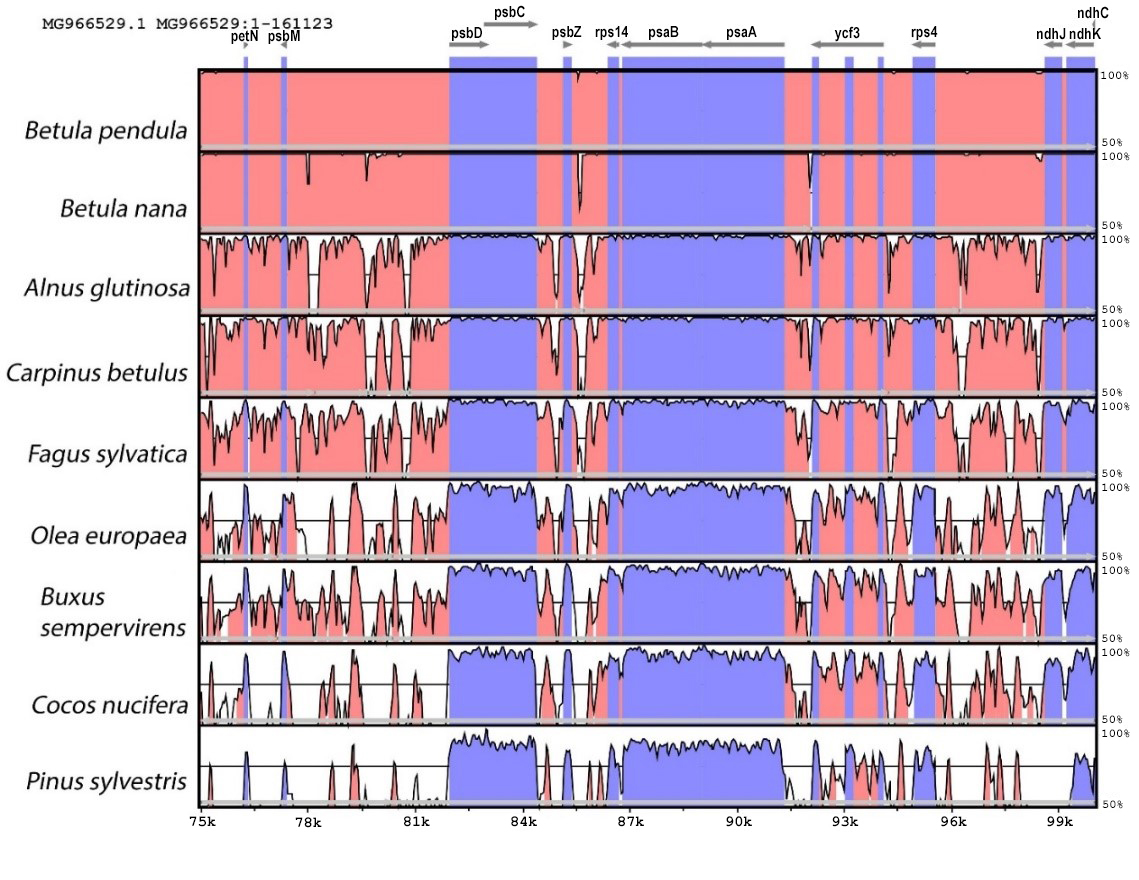 |
| \| 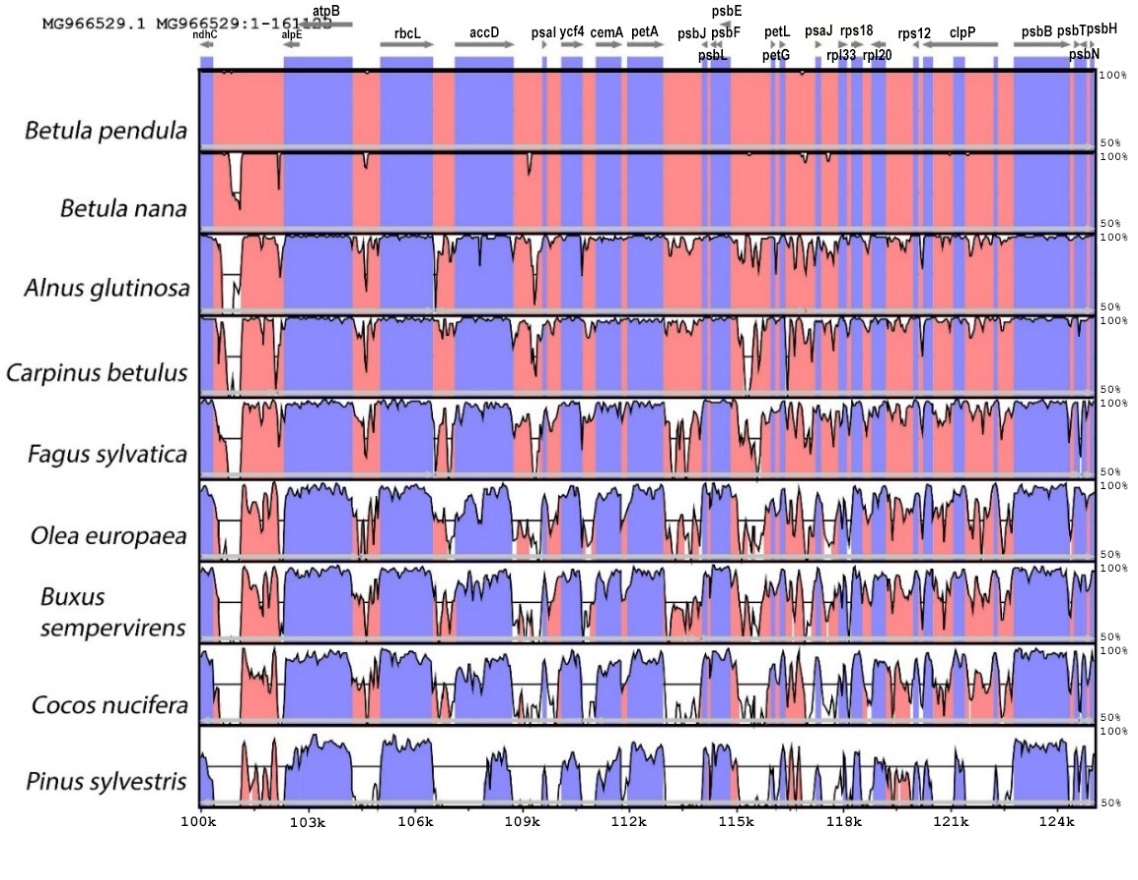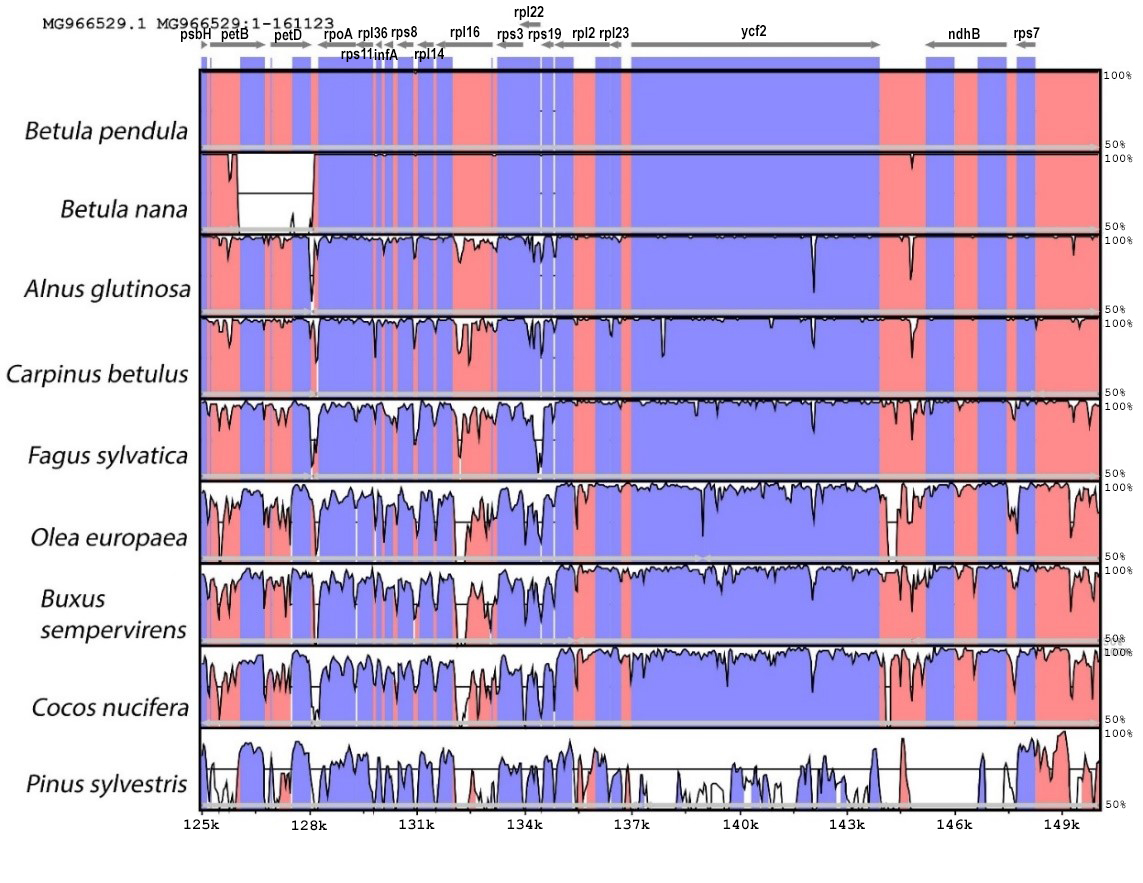 \| \| --- \| \|  \| \| 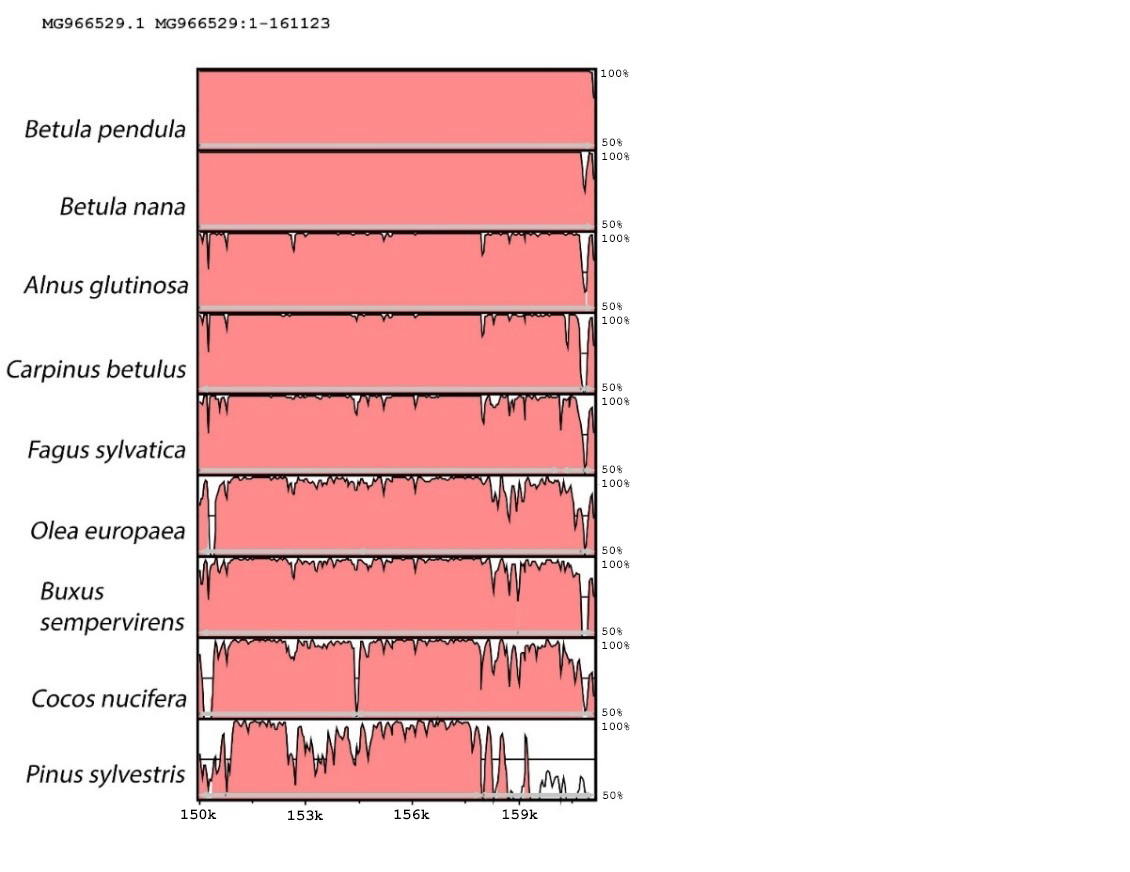 \|   **Supplementary Figure S1.** Multiple nucleotide sequence alignment of nine woody plant species plastomes from *Spermatophyta* (mVISTA). *B. pendula var. carelica* was used as a reference. Grey arrows above the alignment indicate the transcriptional directions of genes. Genome regions are color-coded as exon (violet) and conserved non-coding sequences (CNS) (red). A cut-off of 50% identity was used for the plots. The Y-axis indicates the percent identity between 50 and 100%. |

**
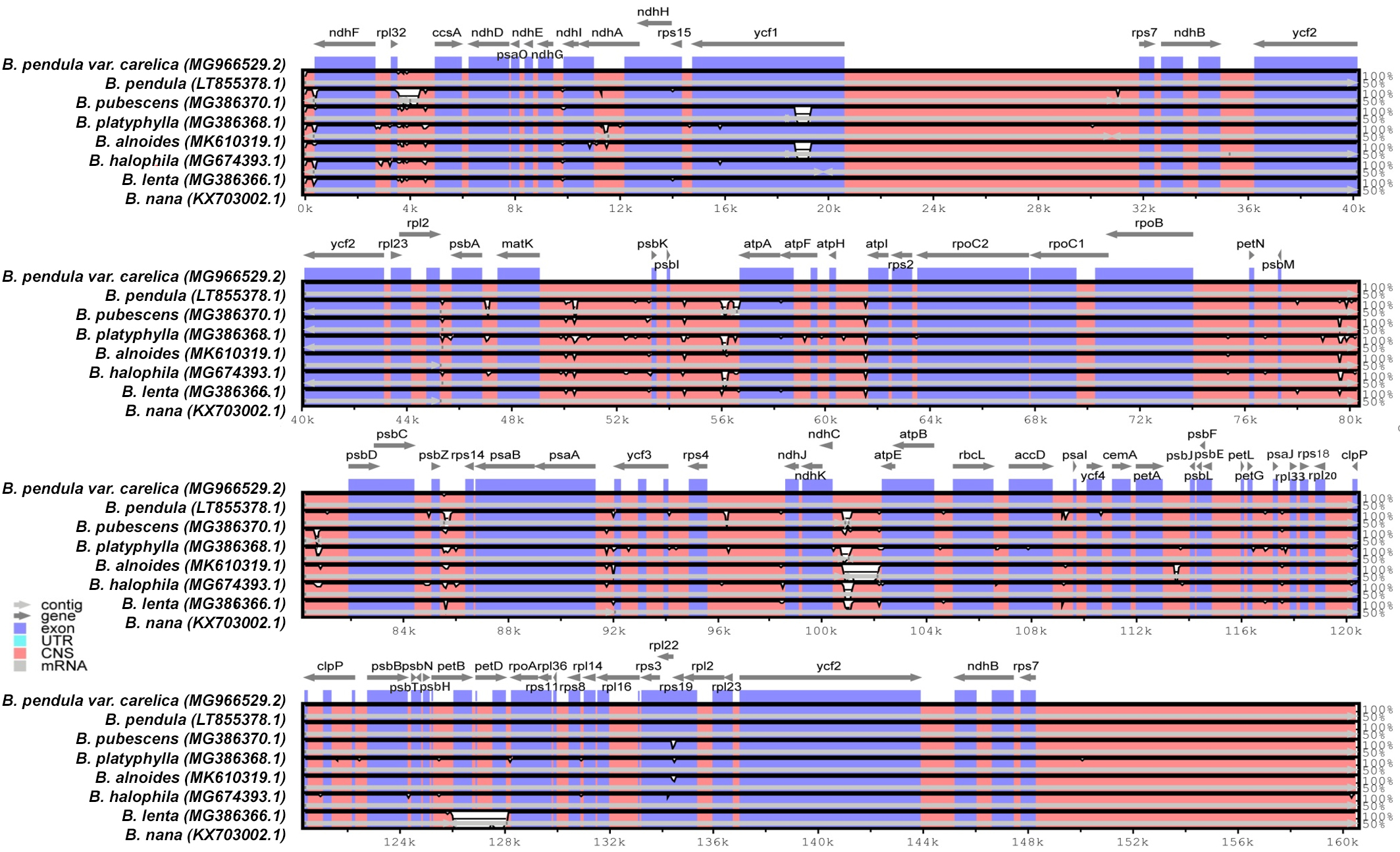
Supplementary Figure S2.** Multiple whole-plastome sequence alignment of eight birch species. Grey arrows above the alignment indicate the transcriptional directions of genes. Genome regions are color-coded.


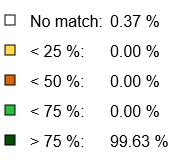

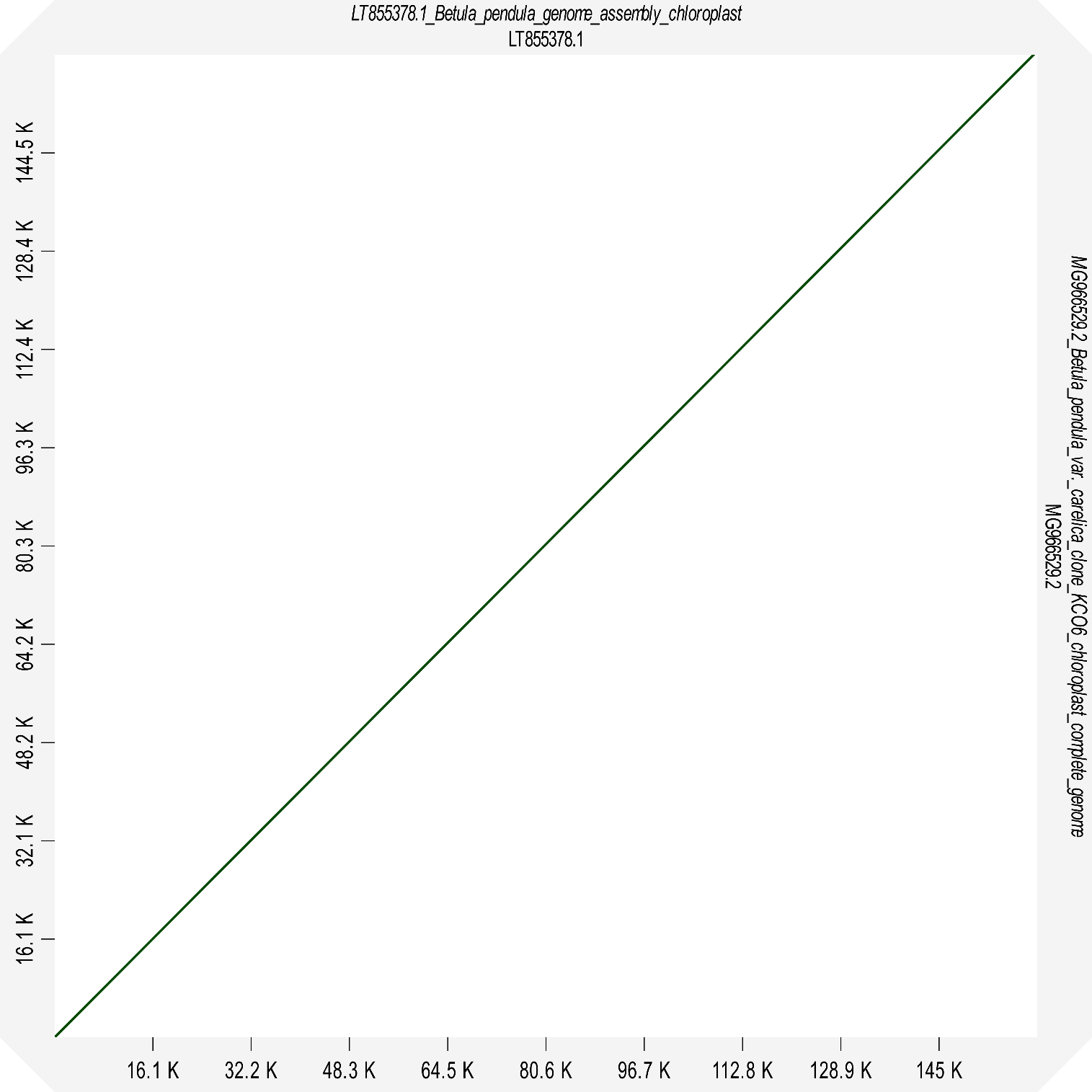


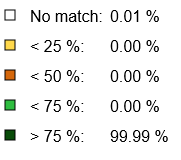

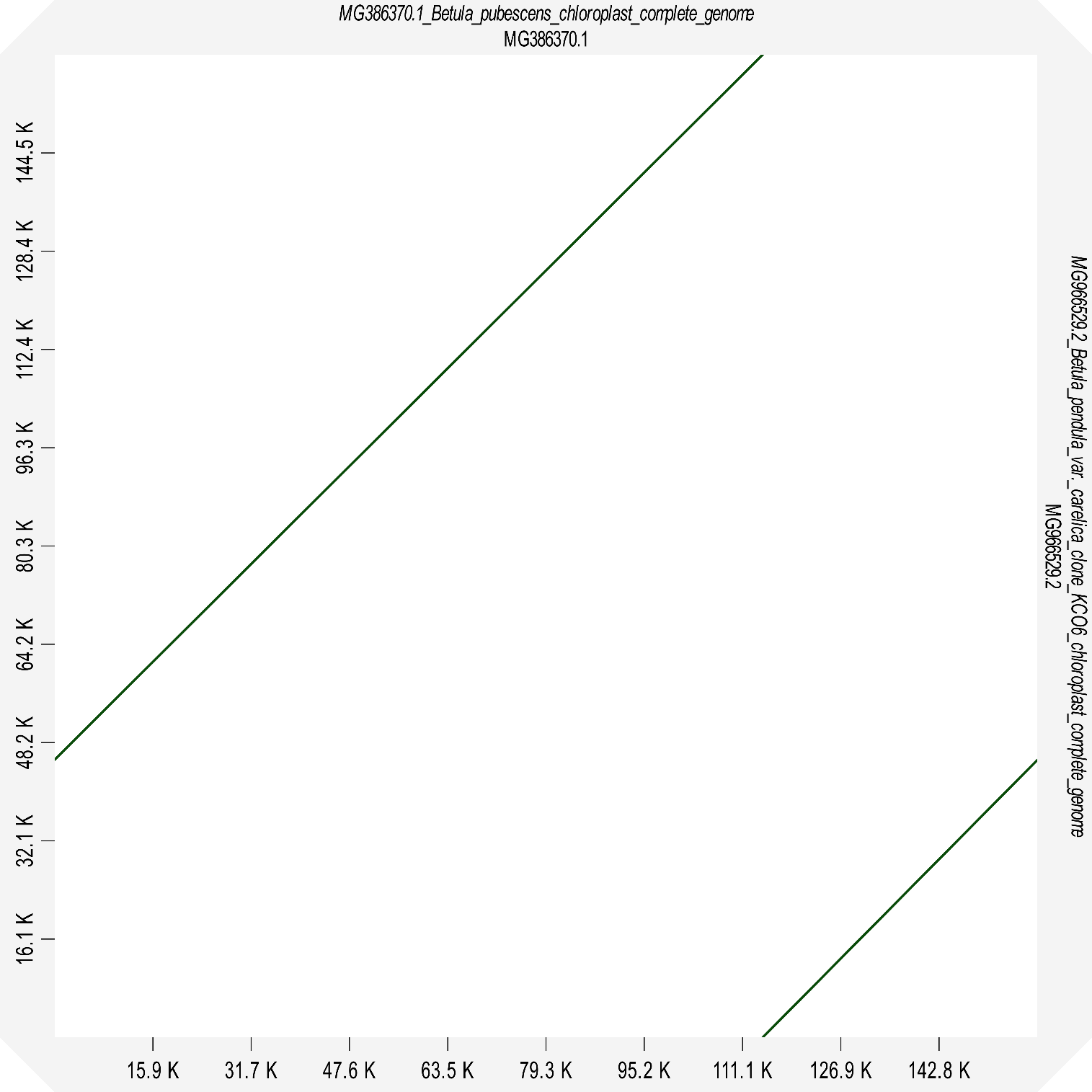


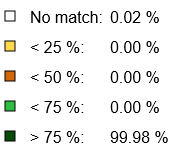

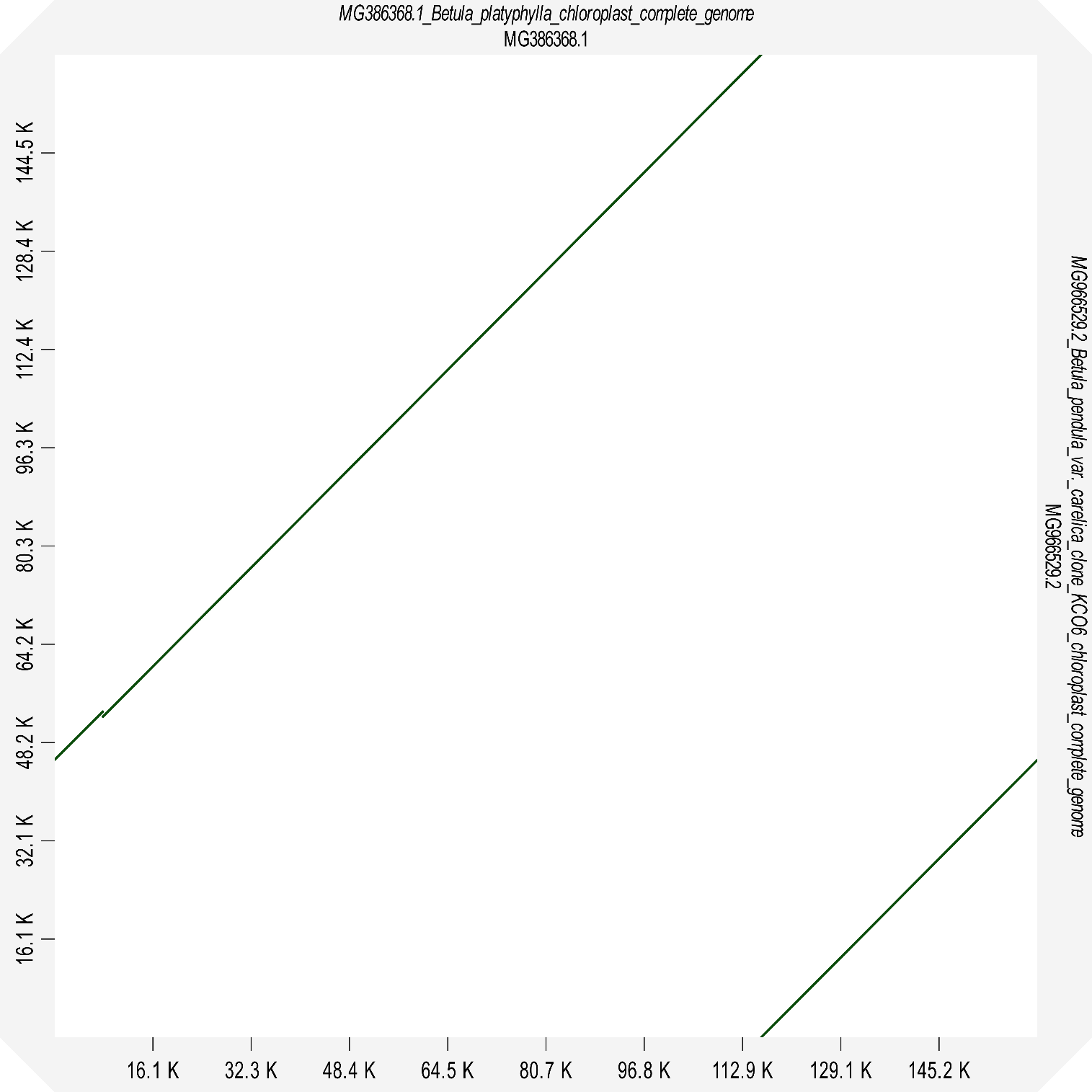


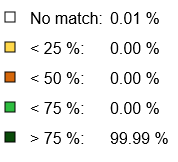

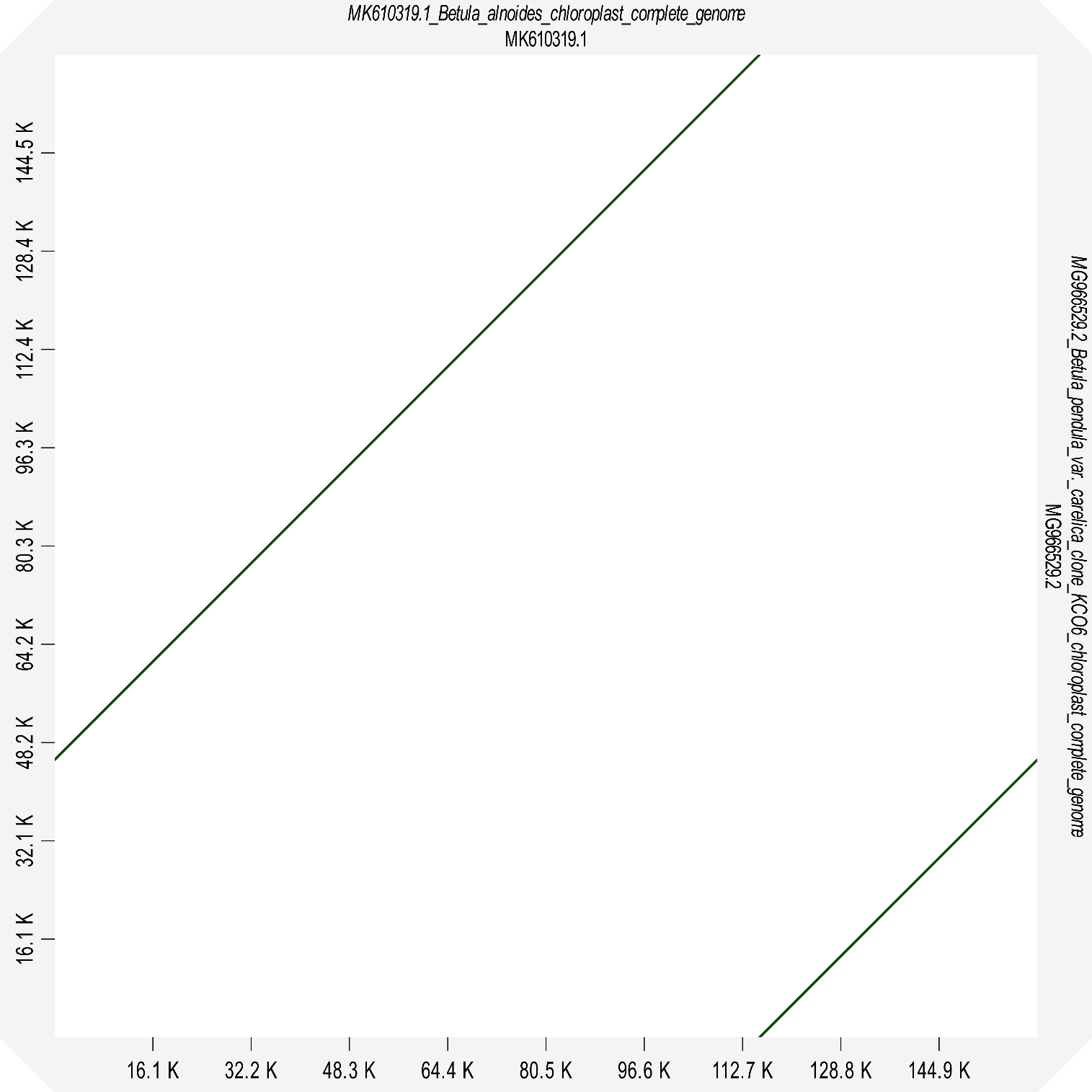


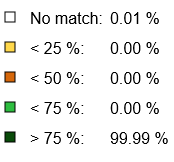

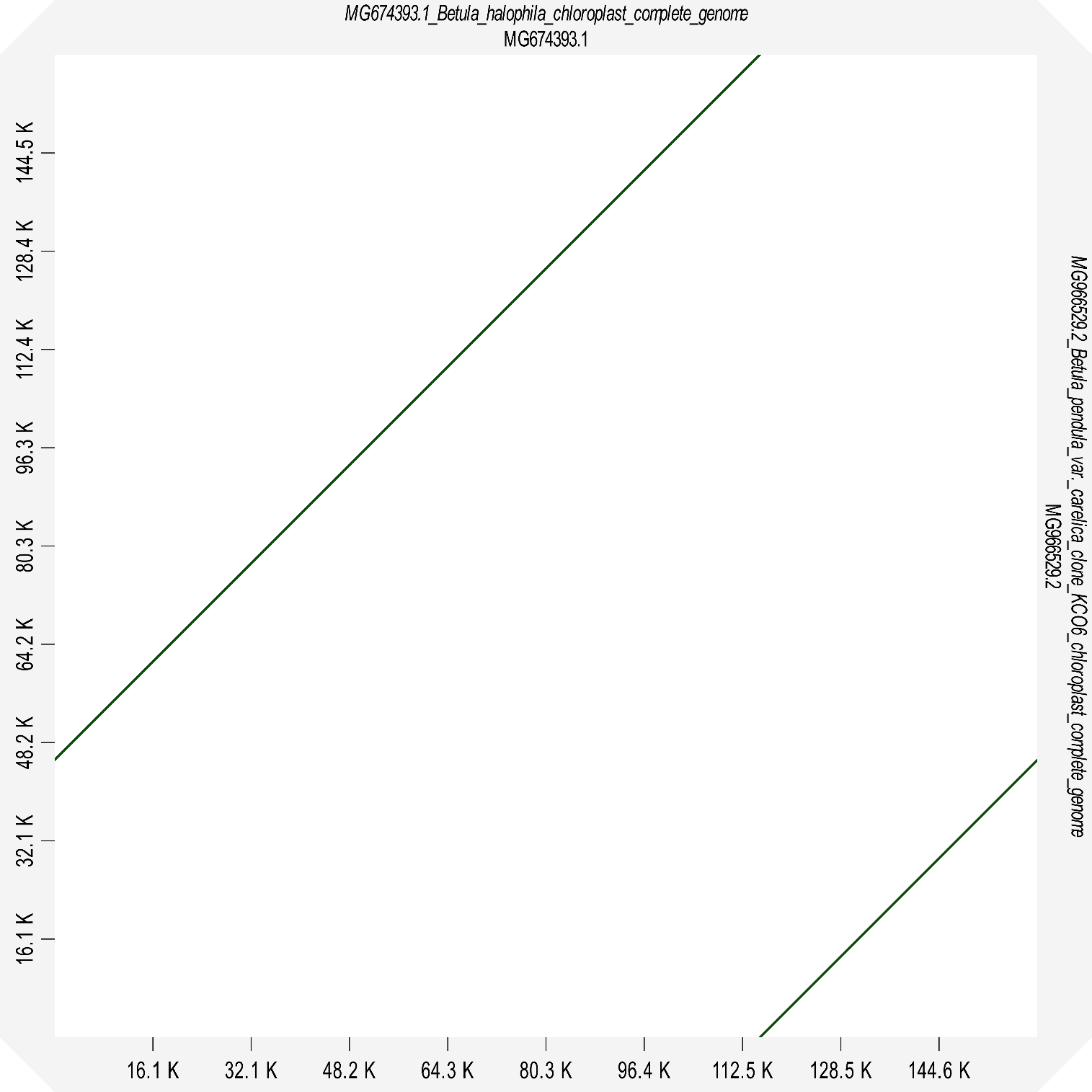


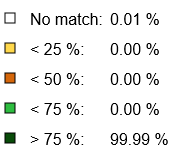

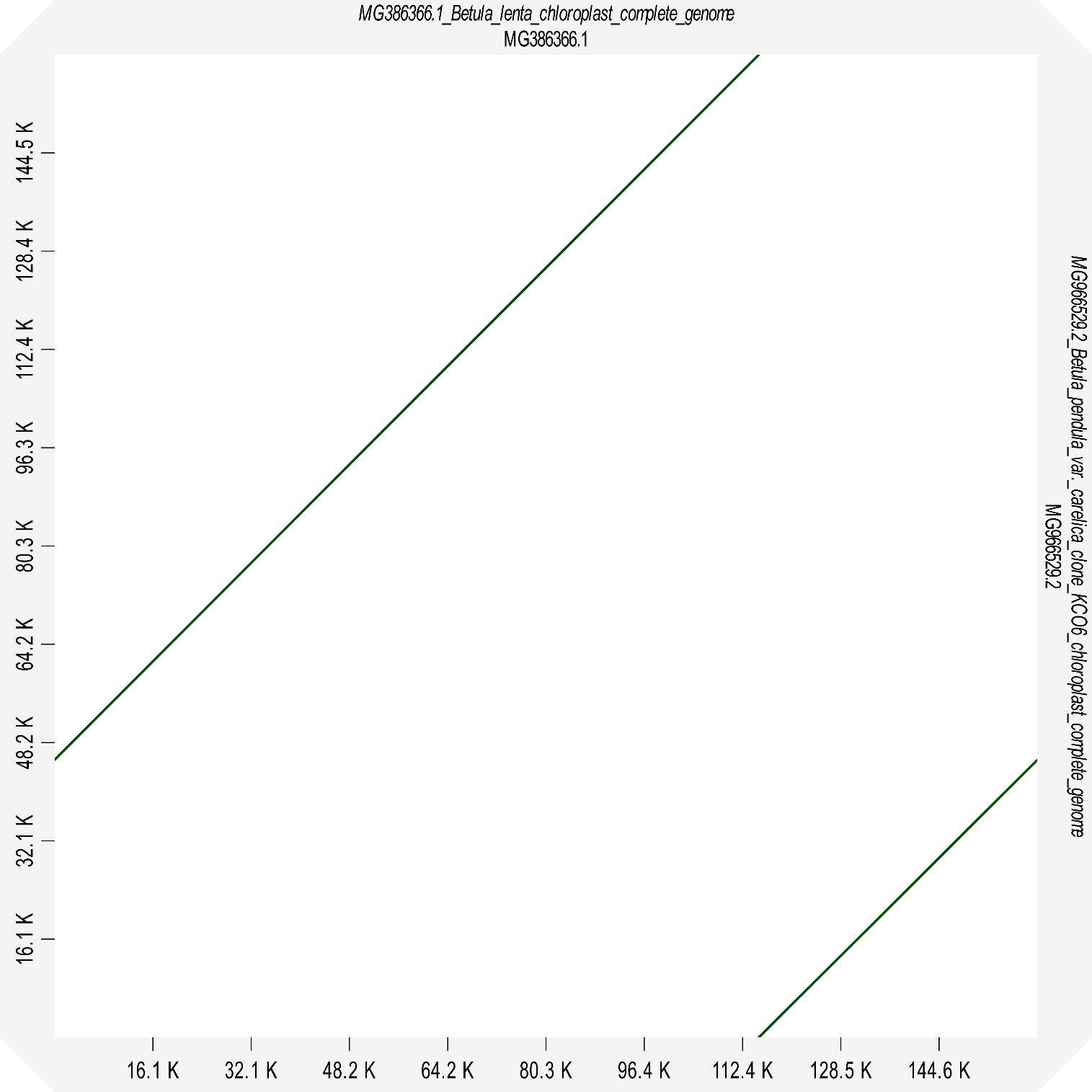


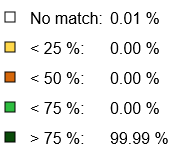

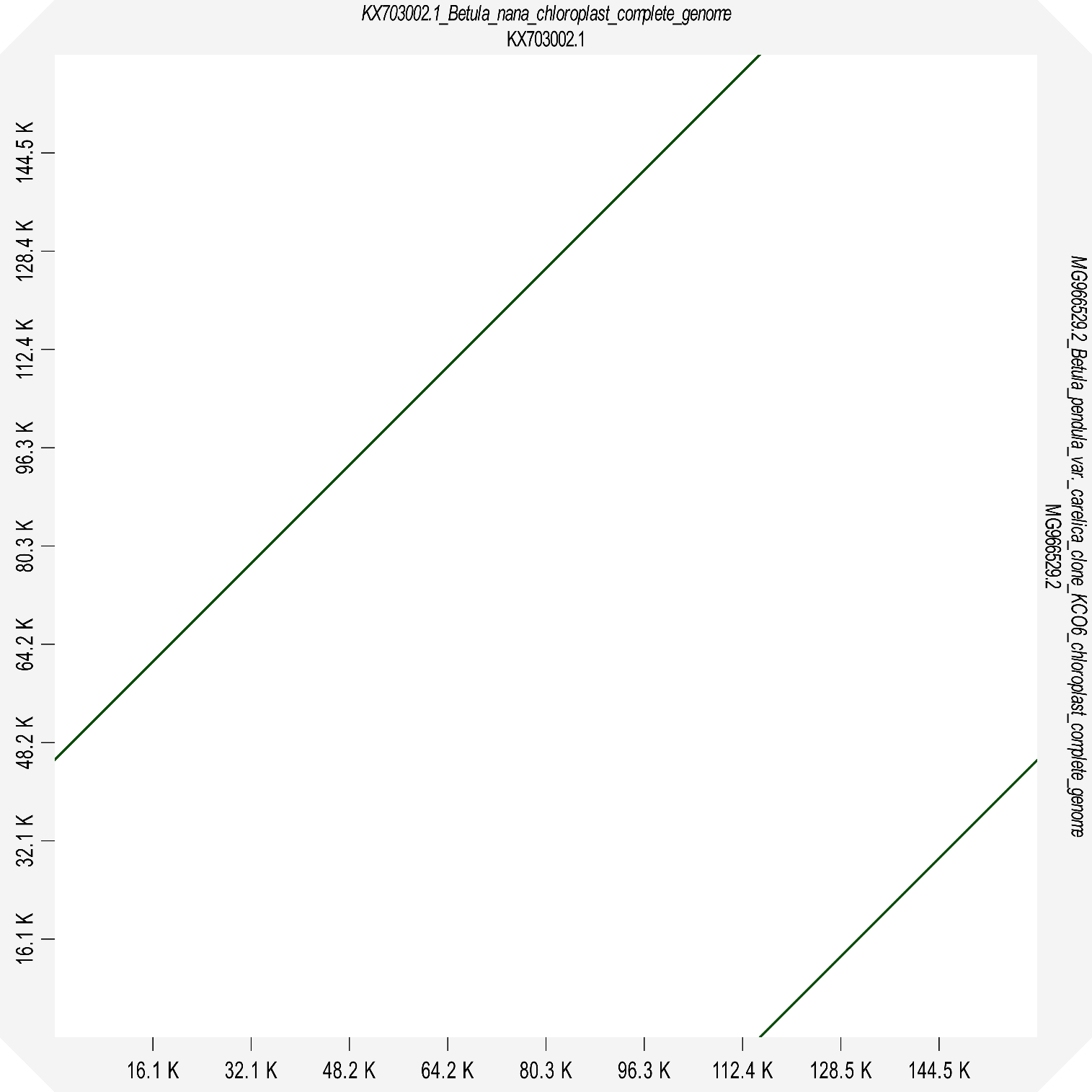


**Supplementary Figure S3.** Dot plots of pairwise alignments of the *B. pendula var. carelica* (MG966529.2) complete plastome sequence vs. plastomes of eight birch species.
